# Striking HIV-1 Entry by Targeting HIV-1 gp41. But, Where Should We Target?

**DOI:** 10.1371/journal.pone.0146743

**Published:** 2016-01-19

**Authors:** Cátia Teixeira, Florent Barbault, Thierry Couesnon, José R. B. Gomes, Paula Gomes, François Maurel

**Affiliations:** 1 Laboratoire Interfaces, Traitements, Organisation et Dynamique des Systèmes–ITODYS–Université Paris Diderot, Paris 7 –CNRS UMR 7086; 15 rue Jean Antoine de Baïf, 75205 Paris Cedex13, France; 2 CICECO–Instituto de Materiais de Aveiro, Departamento de Química, Universidade de Aveiro, Campus Universitário de Santiago, 3810–193 Aveiro, Portugal; 3 UCIBIO-REQUIMTE, Departamento de Química e Bioquímica, Faculdade de Ciências da Universidade do Porto, Rua do Campo Alegre 687, 4169–007 Porto, Portugal; Chinese Academy of Medical Sciences, CHINA

## Abstract

HIV-1 gp41 facilitates the viral fusion through a conformational switch involving the association of three C-terminal helices along the conserved hydrophobic grooves of three N-terminal helices coiled-coil. The control of these structural rearrangements is thought to be central to HIV-1 entry and, therefore, different strategies of intervention are being developed. Herewith, we describe a procedure to simulate the folding of an HIV-1 gp41 simplified model. This procedure is based on the construction of plausible conformational pathways, which describe protein transition between non-fusogenic and fusogenic conformations. The calculation of the paths started with 100 molecular dynamics simulations of the non-fusogenic conformation, which were found to converge to different intermediate states. Those presenting defined criteria were selected for separate targeted molecular dynamics simulations, subjected to a force constant imposing a movement towards the gp41 fusogenic conformation. Despite significant diversity, a preferred sequence of events emerged when the simulations were analyzed in terms of the formation, breakage and evolution of the contacts. We pointed out 29 residues as the most relevant for the movement of gp41; also, 2696 possible interactions were reduced to only 48 major interactions, which reveals the efficiency of the method. The analysis of the evolution of the main interactions lead to the detection of four main behaviors for those contacts: stable, increasing, decreasing and repulsive interactions. Altogether, these results suggest a specific small cavity of the HIV-1 gp41 hydrophobic groove as the preferred target to small molecules.

## Introduction

Conformational folding of biomolecules is a fundamental process as it controls the function of many proteins.[1–4] In the HIV-1 context, it is well known that upon gp120 binding to the host receptor CD4 and chemokine co-receptor CXCR4 or CCR5, gp41 folds from native (non-fusogenic) state to a fusogenic conformation.[5] The formation of this fusogenic structure brings the viral and cellular membranes close together, a necessary condition for membrane fusion to occur. The control of these structural rearrangements is thought to be central to HIV-1 entry and has stimulated strong efforts to develop different strategies of intervention.[6] Therefore, understanding the gp41 conformational mechanism, at the atomic scale, is an important step to plan the fight against the HIV-1 entry into cells.

Structural evidences about the nature of these movements can be obtained by experiments such as X-ray [7] and NMR [8], once these techniques permit to determine the atomic structures of the initial and final states of the transitions. Other experiments (e.g. cryo-electron microscopy [9] and fluorescence resonance energy transfer [10]) give additional information but, generally, the information is not sufficient either to determine the pathway at an atomic level and to fully understand the process. Thus, it is also important to characterize the full folding pathway in order to retrieve information about (i) the forces that drive the process, (ii) the residues involved, (iii) the interactions established, and (iv) the intermediate species formed. This is exactly where computer simulations hold great promise to significantly complement experiments.

There is a real challenge of simulating conformational transition for systems of interest due to the large size and time scales of the process. To enable simulations of such large conformational transitions by molecular dynamics (MD) calculations, many different strategies have been developed to bias the system towards the final state [11]. Among the vast range of methods available, the widely used targeted molecular dynamics (TMD) technique is computationally attractive because the calculation of the constraint is analytical and scales linearly in time and memory with the number of atoms involved.[12, 13] Therefore, in this work we used the TMD method to study the folding pathway of a simplified model of HIV-1 gp41.

**Fig 1:**
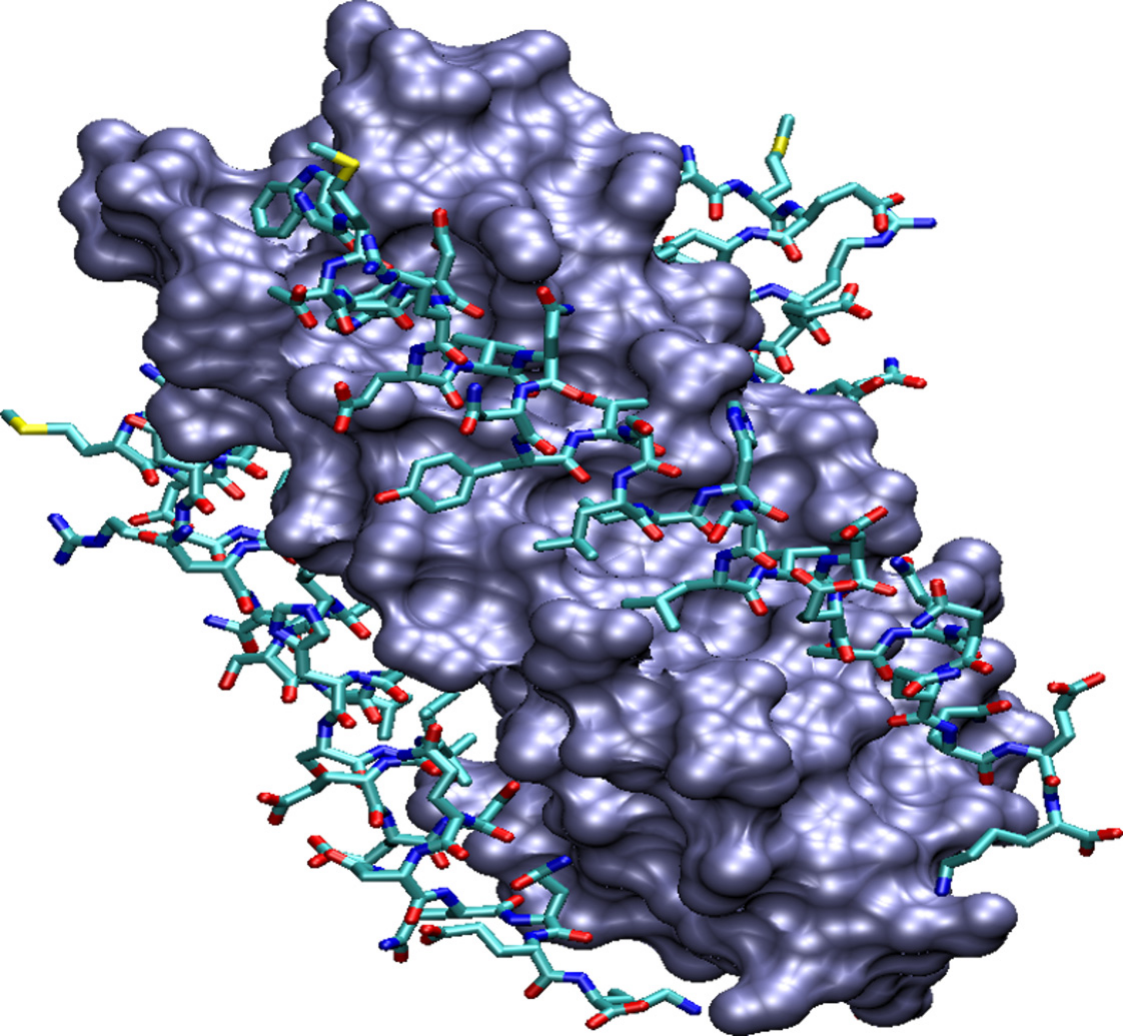
Three-dimensional fusogenic structure of HIV-1 gp41. The C-helices, represented in licorice, are shown against a surface representation of the hydrophobic grooves formed by the N-helices.

Crystallographic studies have revealed a six-helix bundle conformation for the fusion-active gp41.[14, 15] This six-helix bundle can also be described as a trimer-of-hairpins where each hairpin consists of a C-terminal helix packed against an antiparallel N-terminal helix. Three central N-helices form a coiled coil surface with three symmetric hydrophobic grooves, each formed by two adjacent N-helices and allowing C-helix binding (Fig 1).

To explore the structural and mechanistic basis for the helical interactions during the folding process and to limit the size of the system, we have selected the minimal trimeric core of the ectodomain of HIV gp41, N34-(L6)-C28, which comprises residues 546–579 (N34) and 628–655 (C28) of HIV-I Env covalently linked by a six residue SGGRGG loop (L6).[16] In order to reduce time calculation, we took, as a simplified model, 2 N-helices and the C-helix that interacts with such 2 N-helices. Table 1 summarizes the sequence of the HIV-1 gp41 simplified model (the numbering of the residues is provided according to the gp160 sequence). We assumed that this model contains the main interactions that are responsible for the folding process.

**Table 1:** Sequence of residues in the three helices of the simplified model of gp41. The numbering of the residues is provided according to the gp160 sequence. The amino acids found to be important for the conformational change are highlighted in bold. The residues that were not confirmed experimentally are marked with an asterisk.

N2 helix	C helix	N1 helix
Residue number (gp160)	Residue number (gp160)	Residue number (gp160)
^**N2**^**Arg579**		**Arg579**
^N2^Ala578		Ala578
^N2^Gln577		**Gln577**
^**N2**^**Leu576**	**Trp628**	Leu576
^N2^Gln575	Met629	Gln575
^N2^Lys574	Glu630	**Lys574**
^N2^Ile573	**Trp631**	**Ile573**
^N2^Gly572	**Asp632**	Gly572
^N2^Trp571	Arg633	Trp571
^N2^Val570	Glu634	**Val570**
^**N2**^**Thr569**	Ile635	Thr569
^N2^Leu568	Asn636	Leu568
^N2^Gln567	Asn637	Gln567
^N2^Leu566	Tyr638	**Leu566**
^**N2**^**Leu565**	Thr639	Leu565
^N2^His564	Ser640	His564
^N2^Gln563	Leu641	**Gln563**
^**N2**^**Gln562**	Ile642	**Gln562**
^N2^Ala561	His643	Ala561
^N2^Glu560	Ser644	**Glu560**
^**N2**^**Ile559**	Leu645	**Ile559**
^N2^Ala558	Ile646	Ala558
^**N2**^**Arg557**	Glu647*	**Arg557**
^N2^Leu556	Glu648*	**Leu556**
^**N2**^**Leu555**	Ser649	Leu555
^N2^Asn554	Gln650	Asn554
^N2^Asn553	Asn651	Asn553
^N2^Gln552	Gln652	**Gln552**
^**N2**^**Gln551**	Gln653	Gln551
^N2^Gln550	Glu654*	Gln550
^N2^Val549	Lys655*	Val549
^N2^Ile548		Ile548
^N2^Gly547		Gly547
^N2^Ser546		Ser546

**Fig 2:**
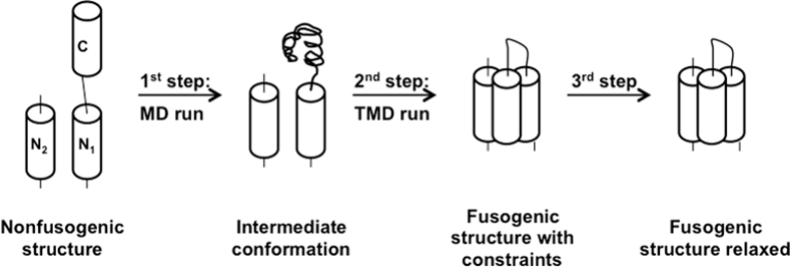
Scheme representing the three-step methodology used for sampling the folding pathways of a simplified model of HIV-1 gp41.

It is well known that the TMD is a powerful method when the initial and final structures are not exceedingly different [17]. Indeed, a first attempt to simulate the conformational transition from the non-fusogenic to the fusogenic form was not successful (data not shown). Hence, we adopted a three-step procedure instead (Fig 2) based on (i) a classical MD simulation starting from the gp41 non-fusogenic structure and leading to an intermediate, followed by (ii) a TMD simulation from the intermediate to the target structure, and by (iii) a relaxation step in order to remove the constraints applied along the targeted trajectory.

The results obtained in this study are going to be discussed and compared to the experimental data obtained from mutagenesis. Several pathways were predicted and a preferred sequence of events emerges when the simulations are analyzed in terms of formations, breakage and evolution of the interactions along the pathway. Additionally, 29 residues were pointed out as predominant ones for the conformational transition, and 2696 possible interactions were reduced to major 48 ones revealing the efficiency of the method. Altogether, these results suggest that targeting the already well-known deep hydrophobic “Trp, Trp-Ile pocket” is, indeed, likely to be the preferred region HIV-1 gp41 hydrophobic groove to be targeted by small molecules and, consequently, to inhibit HIV-1 gp41 conformational change. Additionally, these results also identified key residues and interactions established along the conformational pathway, which will hopefully assist the design of improved HIV-1 gp41 inhibitors and guide experimental researchers that are performing mutagenesis studies.

## Materials and Methods

The preparation of the fusogenic and non-fusogenic structures, and respective minimizations and equilibrium MD simulations are described in Supporting Information (Text A in S1 File). The modifications and protonation of the protein structures were performed with Sybyl [18] software while all minimizations, molecular dynamics, target molecular dynamics and analysis were done with AMBER9. [19] Additional technical details of the three-step procedure are provided in Text A in S1 File, so only a summary of the approach is given below.

### 2.1. Sampling of the folding pathways of a simplified model of HIV-1 gp41

The gp41 non-fusogenic conformation was built by changing the secondary structure of the peptide binding the N- and C-helix from the X-ray structure of the gp41 fusogenic state (PDB code 1DF5). After protonation of the amino acids and minimization of the structure, we performed a classical MD during 1 ns. After this 1ns trajectory, a partial and spontaneous folding of gp41 was observed, which allowed us to verify that the driving force of the process was contained in our simplified model. To predict several pathways, we performed 100 different MD runs (starting from different random seeds) for a total simulation time of 1 ns, converging to different intermediates. The intermediates presenting defined criteria of selection (Text A and Figure A in S1 File) were eligible for the TMD run. The TMD simulation was carried out by slowly decreasing the RMSD at each dynamic step. A force constant of 5 kcal·mol^-1^·Å^-2^ was used and the TMD forces were applied to the Cα atoms of the 3 helices (Val549 to Arg579, Trp628 to Lys655, and ^N2^Val549 to ^N2^Arg579). The targeted RMSD value was linearly decreased, to 0 Å, by increments of 0.1 Å with a time step of 5 ps. The trajectory was saved after each 0.5 ps for further analysis. The relaxation of the final structure was applied to remove the constraint employed along the TMD simulation. The force constant was decreased from 5 to 0 kcal·mol^-1^·Å^-2^ by steps of 25 ps and, after that, the simulation was run for 1 ns.

### 2.2. Data analysis

Two different methods, geometric and energetic, were used to highlight the main interaction established between the residues of the N and C helix. The geometric method (H_b_/E_i_ method), performed by the ptraj module of AMBER9 package, determines the hydrogen bonds and electrostatic interactions along the TMD pathway by keeping track of pair interactions subject to a specified distance (3.5 and 8.0 Å for hydrogen and electrostatic bonds, respectively) and angle cutoff (120° and no angle cutoff for hydrogen bonds and electrostatic interactions, respectively). The energetic method consisted in free energy decomposition on a pairwise per-residue basis using molecular mechanics with a generalized Born/surface area (GBSA) approach (Text A in S1 File). The relative free energy of conformational transition along the TMD pathway, Δ*G*_*CT*_, was estimated by Eq 1:

$$\Delta G_{CT}=\Delta E_{ele}+\Delta E_{vdw}+\Delta G_{p}+\Delta G_{np}$$ (Eq 1)

where Δ*E*_*ele*_ and Δ*E*_*vdw*_ represent the electrostatic and van der Waals terms, respectively, of the gas phase contribution, while Δ*G*_*p*_ and Δ*G*_*np*_ correspond to the electrostatic and non-polar contributions of the solvation free energy, respectively.

#### 2.2.1. Identification of “hot spot” residues. H_b_/E_i_ method

To quantify the importance of each residue, an average percentage of participation (PoP) in hydrogen bonds and electrostatic interactions for all the TMD simulations were calculated according to Eq 2:

$$PoP\left(R_{i}\right)=\frac{\sum_{T=1}^{n}P_{i}}{\sum_{1}^{n}T}\times 100$$ (Eq 2)

where P_i_ refers to the participation indicator at each step of the dynamics: 1 to indicate its participation in hydrogen bond and/or electrostatic interaction and 0 if it is not involved in any interaction. The PoP(R_i_) for the residue R_i_ is calculated by the quotient between the sum of its participation indicators (P_i_) for all TMD simulations and the total number of TMD simulations (T) analyzed. We then assumed that a residue is predominant for the folding process if its PoP(R_i_) > 50%.

**Energetic method:** The percentage of contribution, $PoC_{T_{i}}^{R_{i}}$, of the residue R_i_ to the conformational transition free energy was calculated applying Eq 3:

$$PoC_{T_{i}}^{R_{i}}=\frac{\Delta G_{T_{i}}^{R_{i}}}{\Delta G_{CT}^{T_{i}}}$$ (Eq 3)

where $\Delta G_{T_{i}}^{R_{i}}$ corresponds to the individual contribution of the residue to the free energy and $\Delta G_{CT}^{T_{i}}$ represents the relative conformational free energy of the TMD simulation T_i_. Then, an average percentage of contribution of each residue, PoC(R_i_), to the free energy of the conformational change was calculated. Our study is focused on 90 amino acids, so if each residue has a similar contribution to the free energy, one can estimate that each of them presents an individual contribution of 1,1%. We then assumed that an amino acid would have a predominant contribution to the free energy if PoC(R_i_) > 1,5%.

#### 2.2.2. Identification of the main interactions established by the identified key residues

To determine the leading interactions established by the residues issued from the data analysis described in section 2.2.1, the same methodology was used.

**H**_**b**_**/E**_**i**_ **method:** The percentage of interaction, PoI(R_ij_), between residues, R_i_ and R_j_, was obtained by applying Eq 4:

$$PoI(R_{ij})=\frac{\sum_{T=1}^{n}I_{ij}}{\sum_{1}^{n}T}\times 100$$ (Eq 4)

where I_ij_ represents the interaction indicator: 1 to indicate that an interaction between residues R_i_ and R_j_ is established, and 0 to indicate absence of contact. PoI(R_ij_) is calculated by the quotient between the sum of its interaction indicators (I_ij_) for all the TMD simulations (T) divided by the total number of trajectories analyzed. We then assumed a predominant interaction during the folding process if its PoI(R_ij_) > 50%.

**Energetic method:** The percentage of interaction, $PoI_{T_{i}}^{R_{ij}}$, between two residues, R_i_ and R_j_, for each TMD pathway was estimated with Eq 5:

$$PoI_{{}_{Ti}}^{R_{i,j}}=\frac{\Delta G_{T_{i}}^{R_{ij}}}{\Delta G_{T_{i}}^{R_{i}}}$$ (Eq 5)

where $\Delta G_{T_{i}}^{R_{ij}}$ is the pairwise contribution, of the interaction between R_i_ and R_j_, to the free energy while $\Delta G_{T_{i}}^{R_{i}}$ corresponds to the total individual contribution of residue R_i_ to the free energy. Then, we calculated an average percentage of interaction for each pair of residues, PoI(R_ij_), along all the TMD trajectories.

#### 2.2.3 Evolution of the main interactions over time

The free energy decomposition on a pairwise per-residue basis was also used to infer about the occurrence of specific contacts at a particular moment of the folding pathway. For that purpose, we calculated for all the key interactions identified in 2.2.2. a percentage of interaction by phase, $PoI_{Ph_{k},T_{i}}^{R_{ij}}$, of a pair of residues, R_i_ and R_j_, during a phase (100 ps), Ph_k_, of a TMD simulation, T_i_, applying Eq 6:

$$PoI_{Ph_{k},T_{i}}^{R_{ij}}=\frac{\Delta G_{Ph_{k},Ti}^{R_{ij}}}{\Delta G_{Ti}^{R_{ij}}}$$ (Eq 6)

where $\Delta G_{Ph_{k},T_{i}}^{R_{ij}}$ represents the pairwise contribution, resulting from interaction between residues R_i_ and R_j_, to the free energy during one phase, Ph_k_, of a TMD simulation, T_i_. In turn, $\Delta G_{Ti}^{R_{i},R_{j}}$ corresponds to the total pairwise contribution of the interaction between the same residues (R_i_, R_j_) to the free energy along the total time of the TMD simulation, T_i_. Then, we calculated the average percentage of interaction by phase for each pair of residues, PoI(R_ij_)_Phk_, along all the TMD trajectories. A more detailed description of this procedure is provided as supporting information (Text A and Table A in S1 File).

## Results and Discussion

### 3.1. Selection of the intermediates structures and successful folding pathways obtained by TMD

**Fig 3:**
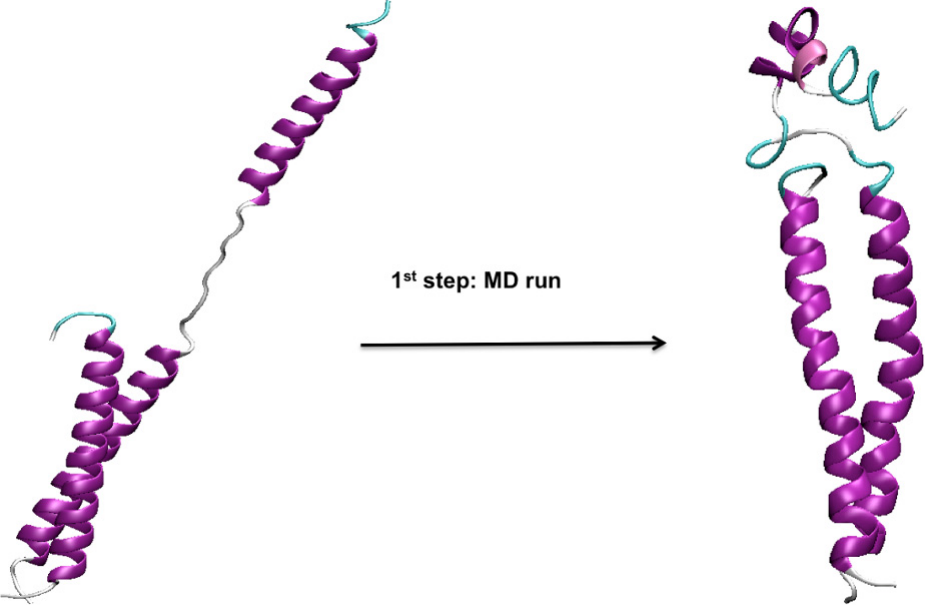
Representation of the first step of the simulation procedure (MD run): the gp41 non-fusogenic conformation (left) evolves to an intermediate structure (right), which will be selected for the second step of the procedure if it complies with defined criteria of selection (Text A and Figure A in S1 File).

Applying the defined criteria of selection (Text A and Figure A in S1 File), 19 of the 100 MD simulations were selected as starting conformations for the TMD runs (Fig 3).

**Fig 4:**
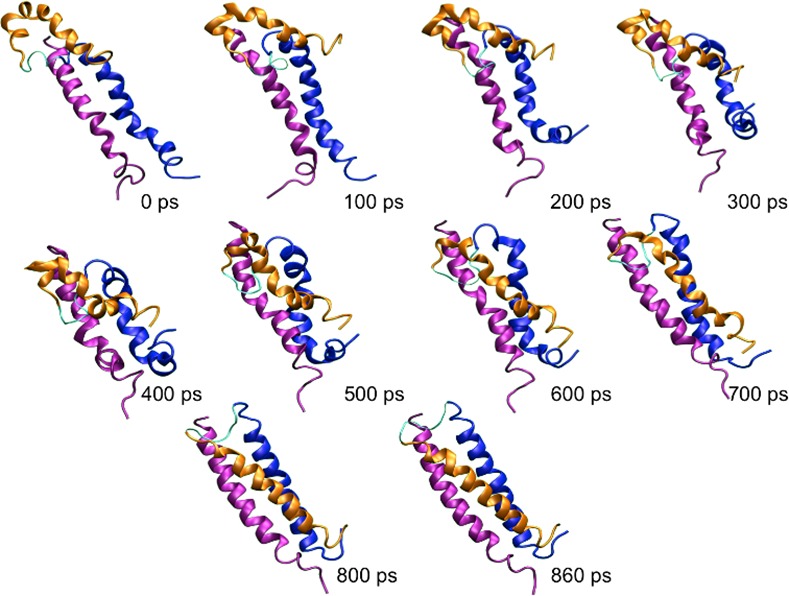
Example of a successful TMD run, representing the convergence of the intermediate structure (0 ps) to the fusogenic conformation (860 ps). The C-helix is represented in orange, while the N1- and N2-helices are represented in blue and purple, respectively.

A visual inspection of the final TMD snapshot allowed us to verify that 14 (74%) TMD simulations reached the final state. Fig 4 represents the sequence of the gp41 conformation along a successful TMD until it converges to the fusogenic structure.

At the end of each TMD simulation, a relaxation step was performed to remove the constraint applied along the trajectory and to determine if the conformation reached during the targeted process was able to remain stable without constraints. We verified that all the conformations, issued from successful TMD runs, were stable during all unrestrained trajectories (data not shown).

### 3.2. Identification of “hot spot” residues

Data analyses using two approaches, geometric (H_b_/E_i_ method) and energetic (free energy decomposition method), were applied to the successful 14 TMD runs following the procedure outlined in section 2.2.1. The results presented here in detail, for both methods, are the outcome of averaging the results over the different pathways.

**Fig 5:**
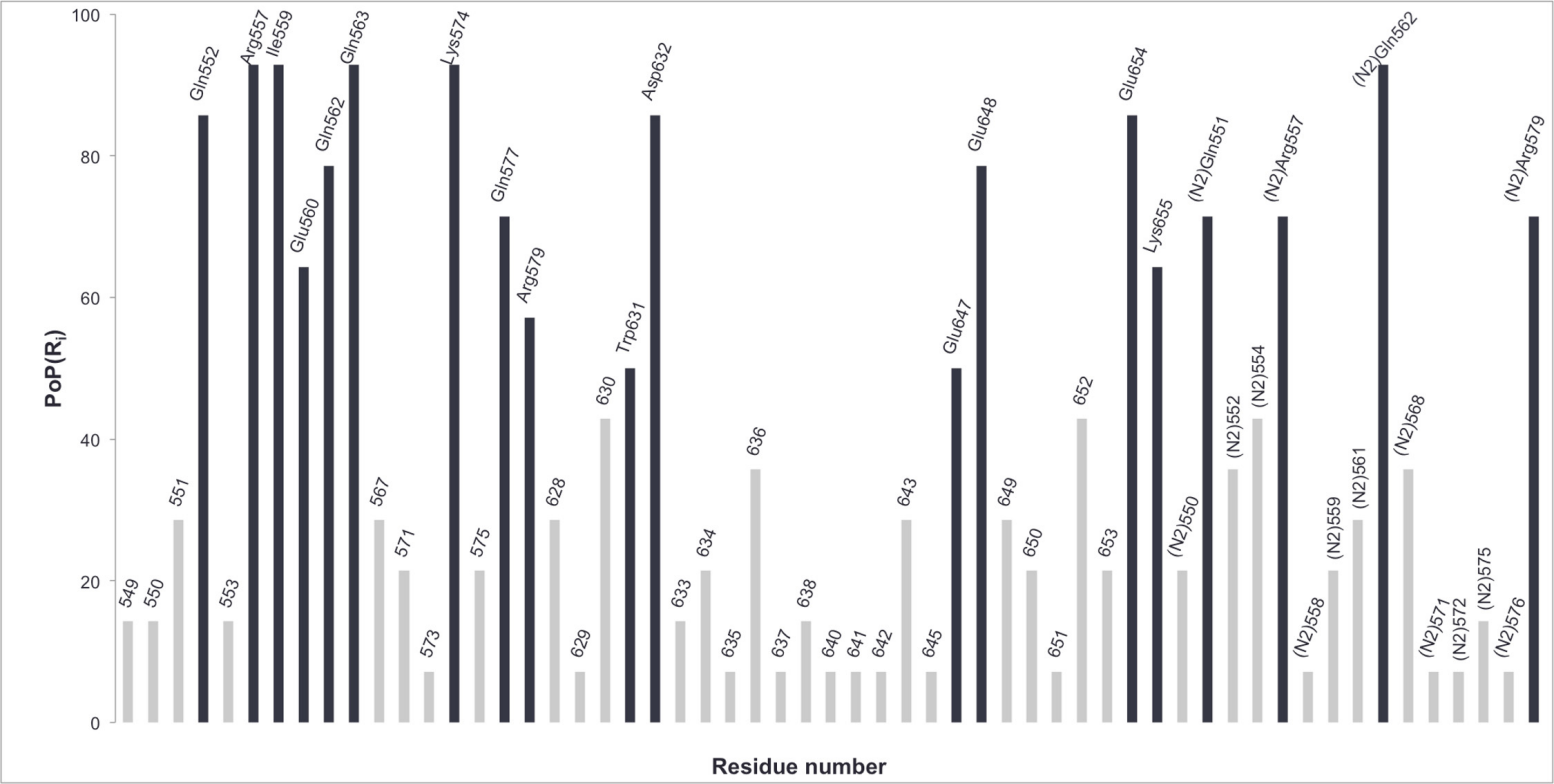
Bar diagram with the values of the percentage of participation for each residue, PoP(R_i_), calculated with the H_b_/E_i_ method. The residues identified with this method as “hot spot” along the HIV-1 gp41 conformational change are represented in black.

Fig 5 displays the average percentage of participation of each residue, PoP(R_i_), in a hydrogen bond and/or electrostatic interaction along the different TMD runs. From this plot, we observe that the amino acids having a major contribution (PoP > 50%) in the folding process correspond to: Gln552, Arg557, Ile559, Glu560, Gln562, Gln563, Lys574, Gln577, Arg579, Trp631, Asp632, Glu647, Glu648, Glu654, Lys655, ^N2^Gln551, ^N2^Arg557, ^N2^Gln562, ^N2^Arg579.

**Fig 6:**
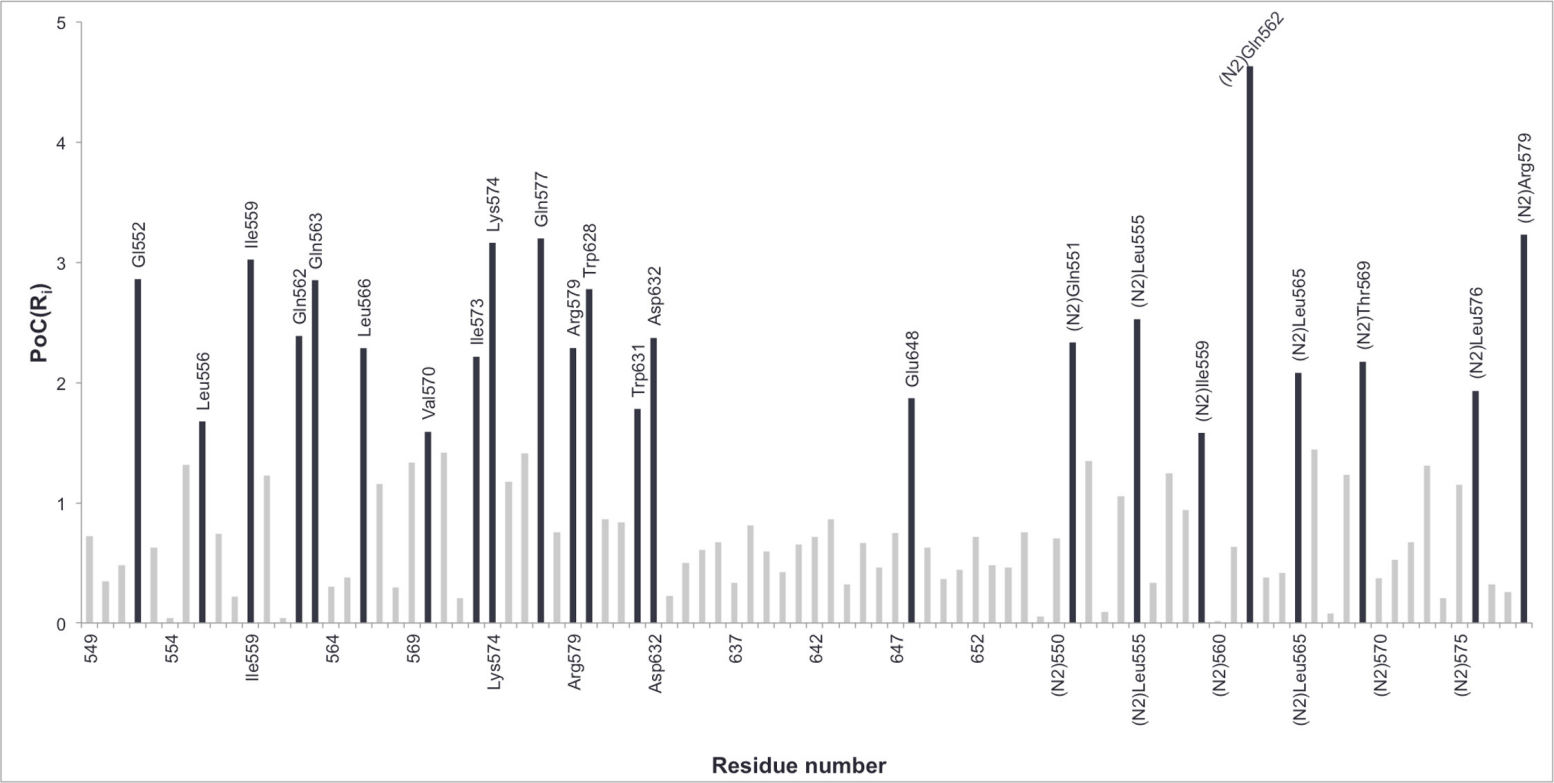
Bar diagram with the values of the percentage of contribution for each residue, PoC(R_i_), calculated with the free energy decomposition method. The residues identified with this method as “hot spot” along the HIV-1 gp41 conformational change are represented in black.

Fig 6 shows the average percentage of contribution of each residue, PoC(R_i_), to the conformational change free energy along the different TMD simulations. From this plot we highlight the amino acids that contribute the most (PoC > 1.5%) to the folding pathway: Gln552, Leu556, Ile559, Gln562, Gln563, Leu566, Val570, Ile573, Lys574, Gln577, Arg579, Trp628, Trp631, Asp632, Glu648, ^N2^Gln551, ^N2^Leu555, ^N2^Ile559, ^N2^Gln562, ^N2^Leu565, ^N2^Thr569, ^N2^Leu576, ^N2^Arg579.

From a global point of view, we can say that the use of both techniques leads to the filtering of the number of residues that are predominant for the conformational transition of HIV-1 gp41. Taking into account both methods, we pointed out 29 residues from the total of 90 amino acids used, corresponding to ~31%. This value represents a significant decrease of the total number of possible interactions. The results show that a larger number of residues belonging to N-helices were identified as key residues when compared to C-helix. This was expected since the N-helices participate, not only in N-C interactions, but also in N-N interactions, so the hydrophobic groove is stabilized.

The results presented above show that the two techniques are complementary because the amino acids that are pointed out by one of the methods only, constitute additional valuable information. They also validate each other because from the 29 residues pointed out as important ones, ~41% were highlighted with both methods. Indeed, each technique estimates the contribution of each residue to the conformational transition taking into account different factors of interaction. The H_b_/E_i_ method is based only on hydrogen bonds and electrostatic interactions that are established along the TMD pathways. The free energy decomposition also estimates, in addition to electrostatic interactions, van der Waals forces and solvation contributions. However, we should keep in mind that the final results of the free energy decomposition analysis correspond to an average along the TMD trajectory. This way we must be aware that if some residues are only involved in interactions at the final phase of the trajectory, their contributions could be underestimated. In the other hand, residues that are crucial for the conformational change, due to van der Waals or solvation contributions, are not taken into account by the H_b_/E_i_ technique. Therefore, we expect that the residues only identified by the H_b_/E_i_ method (e.g. Arg557 or Glu647) are prone to be residues that establish punctual interactions at a specific time of the folding process, while the residues that are only identified by the free energy decomposition (e.g. Val570, Leu566 or ^N2^Thr569) are more prone to mainly establish constant hydrophobic contacts along the entire folding process.

### 3.3. Identification of main interactions

In this section we report the results regarding the main interactions responsible for the HIV-1 gp41 movement. To identify such interactions, data analysis was performed as described in section 2.2.2. The results are an average over the different TMD simulations. The main interactions established by the “hot spot” residues identified by the H_b_/E_i_ and by the free energy decomposition methods are listed in Table 2 and Table 3, respectively.

**Table 2:** Major interactions established by key residues (highlighted in bold) both identified with the Hb/Ei method. The values of the percentage of interaction, PoI(R_ij_), between the two residues are indicated in parenthesis.

"Hot spot" residue (R_i_)	Major interactions identified with the H_b_/E_i_ method
**Gln552**	^**N2**^**Gln551** (80.0); ^N2^Gln552 (33.3); ^N2^Asn554 (25.0)
**Arg557**	**Glu648** (18.2); **Glu654** (100.0); **Lys655** (11.1)
**Ile559**	^**N2**^**Gln562** (100.0)
**Glu560**	Gln652 (33.3); **Lys655** (55.6); ^**N2**^**Arg557** (11.1)
**Gln562**	^N2^Ile559 (27.3); ^**N2**^**Gln562** (90.9)
**Gln563**	**Glu647** (28.6); ^N2^Ala561 (30.8); ^**N2**^**Gln562** (84.6)
**Lys574**	**Asp632** (91.7); **Glu648** (18.2); ^N2^Leu568 (15.4)
**Gln577**	**Asp632** (20.0); **Lys655** (11.1); ^**N2**^**Arg579** (60.0)
**Arg579**	Glu630 (25.0); **Asp632** (50.0); ^**N2**^**Arg579** (37.5)
**Trp631**	^N2^Leu568 (71.4); ^**N2**^**Arg579** (14.3)
**Asp632**	^**N2**^**Arg579** (40.0)
**Glu647**	^**N2**^**Gln551** (14.3); ^**N2**^**Arg557** (57.1); ^**N2**^**Gln562** (14.3)
**Glu648**	**Arg579** (25.0); ^**N2**^**Arg557** (70.0); ^**N2**^**Arg579** (20.0)
**Glu654**	**Lys574** (8.3)
**Lys655**	**Gln552** (11.1); **Lys274** (11.1)
^**N2**^**Gln551**	**Glu648** (10.0)
^**N2**^**Arg579**	Glu630 (40.0)

**Table 3:** Major interactions (attractive: positive values; and repulsive: negative values) established by key residues (highlighted in bold) both identified with the free energy decomposition method. The values of the percentage of interaction, PoI(R_ij_), between the two residues are indicated in parenthesis.

"Hot spot" residue (R_i_)	Major interactions identified with the free energy decomposition
**Gln552**	^**N2**^**Gln551** (36.8); ^N2^Gln552 (28.7); ^**N2**^**Leu555** (18.1)
**Leu556**	^**N2**^**Gln551** (7.1); ^N2^Asn554 (12.3); ^**N2**^**Leu555** (43.6)
**Ile559**	^**N2**^**Leu555** (14.0); ^**N2**^**Ile559** (22.4); ^N2^Gln562 (41.9)
**Gln562**	^**N2**^**Ile559** (12.5); ^N2^Gln562 (60.2); ^N2^Leu566 (12.9)
**Gln563**	^N2^Ala561 (9.7); ^N2^Gln562 (45.7); ^**N2**^**Leu565** (5.9)
**Leu566**	^N2^Gln562 (17.5); ^**N2**^**Leu565** (29.3); ^N2^Leu566 (26.5)
**Val570**	^**N2**^**Leu565** (18.5); ^N2^Leu568 (13.4); ^**N2**^**Thr569** (31.1)
**Ile573**	^**N2**^**Thr569** (18.8); ^N2^Ile573 (24.5); ^**N2**^**Leu576** (18.1)
**Lys574**	**Trp628** (9.9); **Trp631** (9.1); **Asp632** (19.3)Arg633 (-1.6); ^N2^Lys574 (-2.3); ^**N2**^**Arg579** (-2.7)
**Gln577**	**Trp628** (9.5); ^**N2**^**Leu576** (20.8); ^**N2**^**Arg579** (24.3)
**Arg579**	Glu630 (16.8); **Asp632** (19.5); Glu634 (12.8)Arg633 (-6.5); ^N2^Lys574 (-8.1); ^**N2**^**Arg579** (-3.6)
**Trp628**	**Gln575** (8.5); **Arg579** (13.0); ^**N2**^**Arg579** (15.6)
**Trp631**	Trp571 (13.9); ^**N2**^**Arg579** (11.4)
**Asp632**	^**N2**^**Arg579** (15.2); Glu560 (-3.4); ^N2^Glu560 (-3.1)
**Glu648**	Arg557 (14.1); ^N2^Arg557 (20.5); ^**N2**^**Arg579** (10.9)Glu560 (-4.5); ^N2^Glu560 (-3.0)
^**N2**^**Gln551**	Val549 (12.3); Asn553 (11.1)
^**N2**^**Leu555**	Leu555 (24.4)
^**N2**^**Ile559**	Leu555 (11.8)
^**N2**^**Leu565**	Gln567 (9.0)
^**N2**^**Thr569**	Thr569 (27.9)
^**N2**^**Leu576**	**Arg579** (9.9); Leu576 (30.9)
^**N2**^**Arg579**	Glu630 (11.8); Arg633 (-5.4)

From Table 2 we can observe that, in general, each residue is found to interact predominantly with one specific residue (e.g. Lys574 with residue Asp632; and Trp631 with residue ^N2^Leu568). However, in some cases (e.g. Gln552 and ^N2^Arg579) the interaction is less specific and the residues establish several additional contacts. From Table 3, the same tendency is verified, i.e. each key residue is inclined to predominantly interact with one specific residue. Still, this overall feature is not as well defined as for the H_b_/E_i_ technique. For example, the residue Ile559 is found, with the H_b_/E_i_ method, to predominantly interact with ^N2^Leu568. However, the results from the free energy decomposition analysis indicate that Ile559 also makes important contacts with other amino acids such as ^N2^Leu555 and ^N2^Ile559. As mentioned above, this was expected as the analysis performed by free energy decomposition also takes into account van der Waals interactions and a hydrophobic surface area term.

Some key residues establish interactions with particular features, which will be discussed here in more detail. For the remaining main interactions established by each key residue, a detailed description is provided in Text B in S1 File. The description is made by taking into account the analysis performed by both H_b_/E_i_ and free energy decomposition methods, so we can directly compare the information provided by each technique.

#### Lys574

This amino acid was outlined by the two used methods, and both identified a strong interaction with **Asp632** (salt bridge between the side chains). This interaction seems to be crucial for the stability of the trimer-of-hairpins since it corresponds to a contact between the N-C helices. From the free energy decomposition method, we also observed significant interactions with two more residues belonging to C-helix, namely with **Trp628** and **Trp631**. These contacts are thought to participate in the stabilization of the C-helix upon its accommodation into the hydrophobic groove formed by N-helices. Additional interactions were also detected with another C-helix amino acid, **Glu648**. This residue is not spatially adjacent to Lys574, which let us think that lysine, in addition to promote the stability of the trimer-of-hairpins, could act as an “anchor” point to boost the movement of the C-helix towards the target conformation. Based on the results, a third function could be assigned to Lys574 due to the interaction that it establishes with ^**N2**^**Leu568**, suggesting that this contact could also be involved in the stabilization of the hydrophobic groove. Finally, we also observed the occurrence of repulsive interactions established with some residues such as ^**N2**^**Arg579** and ^**N2**^**Lys574**. The relevance of these repulsive contacts will be discussed in Section 3.4.

#### Gln577

This amino acid was highlighted by both methods and presents a double function since it establishes interactions with C-helix (**Asp632**) and N-helix residues (^**N2**^**Gln575**, ^**N2**^**Leu576** and ^**N2**^**Arg579**). The contacts formed with the amino acid belonging to C-helix suggest that Gln577 contributes to the formation and stability of the trimer-of-hairpins, while the interactions made with N-helix residues show that **Gln577** is also involved in the stability of the hydrophobic groove.

#### Arg579

This residue is pointed out by the two techniques and, as observed for Lys574, it appears to be involved in three distinct tasks. The first one is the stability of the hydrophobic groove evidenced by the interaction that it establishes with ^**N2**^**Leu576**. The second task is its contribution to the formation of the trimer-of-hairpins since contacts with C-helix residues (**Trp628**, **Glu630** and **Asp632)** were observed. The third function of Arg579 is that it also acts as an anchor point, just like Lys574, promoting the movement of the C-helix toward its final conformation by interacting with C-helix residue **Glu648**, that does not belong to its neighbourhood in the fusogenic structure. The same repulsive interactions detected for Lys574 were also observed for Arg579.

#### Trp628

This amino acid was outlined with the free energy decomposition analysis. The favourable contribution to the conformational transition free energy is mainly due to the significant interactions that this residue establishes with ^**N2**^**Arg579**, **Gln577**, **Lys574**, **Gln575** and **Arg579**. The contacts made with the first three residues are crucial for the stability of the trimer-of-hairpins formation. Indeed, all the three amino acids are spatially adjacent to Trp628 and, therefore, create van der Waals interactions with it. Arg579 and Gln575 are more distant from the neighbouring environment of Trp628, suggesting that they may be involved in an “anchor” function during the large movement of gp41 by establishing electrostatic interactions.

#### Glu648

Both techniques put this residue in evidence. The results show that it predominantly interacts with ^**N2**^**Arg557** but it also presents significant interactions with **Arg557**, **Lys574**, **Arg579** and ^**N2**^**Arg579**. The electrostatic interactions formed with ^N2^Arg557 and Arg557 are thought to contribute to the stability of the trimer-of-hairpins. The contacts observed with ^N2^Arg579, Arg579 and Lys574 are anchor points so the C-helix keeps the right direction during its movement towards the final conformation. Repulsive interactions with Glu560 and ^N2^Glu560 were observed.

#### ^N2^Gln562

This residue was outlined by the two methods. Three strong electrostatic interactions were observed with **Ile559**, **Gln562** and **Gln563**. These results suggest that this interaction strongly participates in the stability of the hydrophobic groove.

#### ^N2^Arg579

This amino acid was highlighted by the two techniques and interactions with several residues were observed. Similarly to what we observed for Lys574 and Arg579, ^N2^Arg579 is a “multi-task” residue. A main interaction with **Gln577** is observed, which is thought to mostly contribute to the stability of the hydrophobic groove. We also detected contacts with the C-helix amino acids **Trp628, Glu630** and **Asp632,** which stabilize the formation of the trimer-of-hairpins. Additionally, the “anchor point” function of ^N2^Arg579 is due to the interaction formed with Glu648, an amino acid that is not on its neighbourhood once the large movement of gp41 is completed. And, finally, repulsive contacts with Lys574, Arg579 and Arg633 were also observed.

### 3.4. Evolution of main interactions along time

The determination of the evolution of the main interactions during the simulation allowed us to have a general view about the interactions that are made and broken within the pathway, and showed us how the movements are transferred from one residue to the others. Such data analysis was performed for all the key residues identified by both H_b_/E_i_ and free energy decomposition methods, following the procedure described in Section 2.2.3. The results presented here are average results over the different TMD simulations.

**Fig 7:**
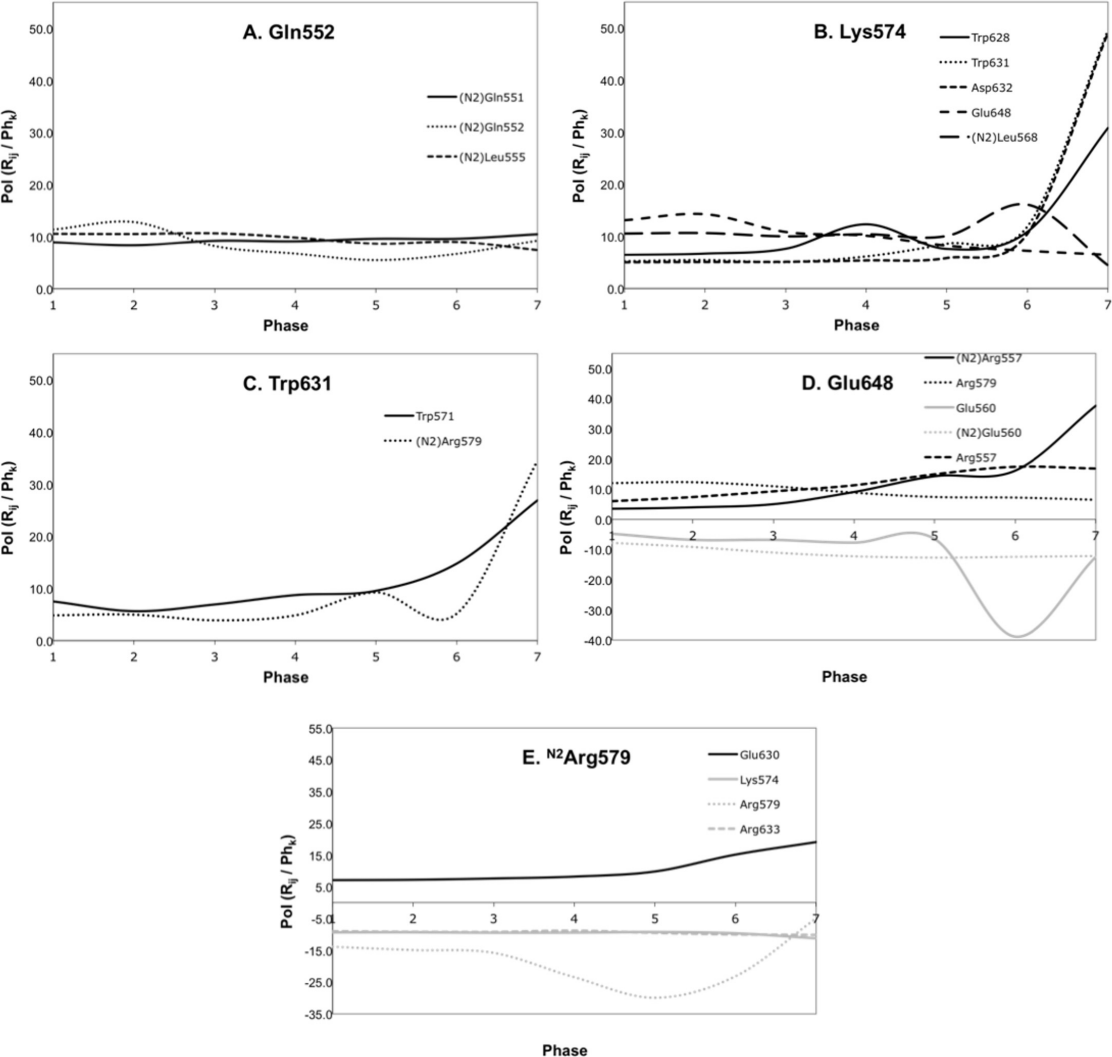
The different types of interactions found along the HIV-1 gp41 conformational change are exemplified with the plots of the evolution of the main interactions identified for residues Gln552, Lys574, Trp631, Glu648 and ^N2^Arg579.

Fig 7 exemplifies the different types of interactions found along the conformational changes.

We observed four main behaviors for the interactions analyzed:

#### Stable interactions (pillars)

These contacts present values of the percentage of interaction between residues R_i_ and R_j_, PoI(R_ij_), that remain constant along the different phases (Ph_k_) of the average TMD pathway (e.g. interaction between Gln552 and ^N2^Gln551; Fig 7A). These interactions are mainly established by residues belonging to the two N-helices and are distributed along the α-helix axis. Therefore, these contacts are thought to be responsible for maintaining the N-helices close together by conferring high stability to the hydrophobic groove. In addition, we also verified that residues from N-helices establish hydrophobic contacts with some amino acids of the C-helix after its accommodation into the hydrophobic groove. Indeed, when we represent the surface of the residues that match this class of interactions, we easily see that they form the floor of the hydrophobic groove (Fig 9 in yellow). Thus, based on these results we can say that the hydrophobic contacts that contribute to maintain the trimeric structure are established between C-helix residues and the N-helices residues that form the floor of the cavity. These results suggest it is crucial that the hydrophobic groove is well defined, presenting a high stability, so the C-helix can be able to fit into. Analogically speaking, we can say that these stable interactions are the “pillars” of the hydrophobic groove.

#### Increasing interactions (locks/guides)

These interactions present a PoI value that increases along the average TMD trajectory (e.g. interaction between Lys574 and Trp631; Fig 7B). In general, the main interactions that fit this class correspond to contacts between residues from N- and C-helices. This class is that presenting the higher number of interactions. Some considerations should be made for increasing interactions:

1. Two distinct growths are noted, sharp and continuous. The predominant increment observed, the sharp one, corresponds to a sudden increase that mostly appears in the last 200 ps (e.g. Lys574-Asp632; Fig 7B), while the second type of increment, the continuous, is performed in a more regular way along the TMD pathway (e.g. Glu648-Arg557; Fig 7D).
2. The sharp type has the particular feature to become very strong for the last 200 ps. The contacts are mostly established between N-helices and C-helix, which permit us to hypothesize that they are involved in the stability of the trimer-of-hairpins. Analogically speaking, we see them as the “locks” of the trimeric structure.
3. The interactions presenting a continuous growth are probably major contacts responsible for leading the C-helix towards its final conformation. All the residues involved are charged, suggesting that the movement of the C-helix is guided by long-range electrostatic interactions. Analogically speaking, we see them as the “guides” of HIV-1 gp41 folding.

#### Decreasing interactions (anchors)

These contacts present a decrease of PoI value as the intermediate moves towards its final conformation (e.g. interaction between Lys574 and Glu648; Fig 7B). With just a single exception, the interactions that fit this class involve three specific N-helices residues (^N2^Arg579, Arg579 and Lys574) and two residues belonging to the C-helix (Glu648 and Glu654). But, more interesting, is that these contacts are created between residues that are not in the same neighborhood in the fusogenic structure. The residues from C-helix, and involved on the decreasing interactions, were also found to be involved in some increasing interactions. Indeed, we observed that some contacts of the two classes of interactions are concerted. As the attractive increasing interactions between residues from C-helix and low residues from N-helices are getting stronger, the decreasing contacts between the same amino acids from C-helix and upper residues from N-helices are getting weaker. This suggests that prior to the attraction of the C-helix, towards the bottom of the hydrophobic groove, the C-helix is anchored at the top of the N-helices. Thus, analogically speaking, we designated the three residues from N-helices involved in decreasing interactions as “anchor points”.

#### Repulsive interactions (retractors)

These contacts present a negative value of PoI, which means unfavorable contacts between the residues (e.g. interaction between ^N2^Arg579 and Arg579; Fig 7E). Some repulsive interactions between charged residues are observed all along the TMD pathway and seemed to be predominant for the conformational change of gp41. According to their type, we can conceive different possibilities for the implication of these interactions:

1. we observed a first and predominant type that corresponds to repulsive interactions between residues from N-helices (e.g. ^N2^Arg579-Arg579). We hypothesize that these contacts are significant for the conformational pathway by avoiding the drawdown of random residues over the hydrophobic groove. For example, if there is no repulsive interaction between ^N2^Arg579 and Arg579, any residue could create interactions that would direct it over the cavity and, hence, it would contribute to the blockage of the hydrophobic groove. Therefore, we think that these interactions are important in maintaining the cavity free for the accommodation of the C-helix.
2. the second type of repulsive interactions is observed between residues from N- and C-helices (Glu648-^N2^Glu560; Fig 7D) and we suppose that they are aimed to help the C-helix to not stuck into the cavity at the halfway of the conformational change. This is the case of the repulsive contacts observed for Glu560-Glu648 and ^N2^Glu560-Glu648. If such repulsive interactions did not exist, C-helix could accommodate into the hydrophobic groove at the level of Glu560 and ^N2^Glu560, by establishing interactions through Glu61 with other residues of the N-helices, instead of proceeding towards the target structure.

Overall, these results suggest that repulsive contacts are used to maintain a certain distance between specific residues (especially between the charged ones) in order to avoid the obstruction of the cavity and that the C-helix is trapped at a particular conformation during the conformational change pathway. Analogically speaking, we see them as “retractors”.

The most significant interactions highlighted in Section 3.3 were classified according to its evolution along time as summarizes in Table 4. The respective plots are available in Figure B in S1 File.

**Table 4:** The leading interactions distributed into the different classes, according to their pattern of evolution along the TMD simulation.

**Stable interactions**	Gln552-^N2^Gln551 / Leu556-^N2^Leu555 / Ile559-^N2^Gln562 / Gln562-^N2^Gln562 / Gln563-^N2^Gln562 / Leu566-^N2^Leu565 / Val570-^N2^Thr569 / Ile573-^N2^Leu576 / Gln577-^N2^Leu576 / Arg579-Glu630 / Arg579-Asp632 / Arg579-Glu634 / Asp632-^N2^Arg579 / Ile559-^N2^Ile559
**Increasing interactions**	Arg557-Glu648 / Arg557-Glu654 / Glu560-Lys68 / Glu15-Gln652 / Lys574-Asp632 / Lys574-Trp628 / Lys574-Trp631 / Gln577-Trp628 / Gln577-^N2^Arg579 / Arg579-Trp628 / Trp628-^N2^Arg579 / Trp631-^N2^Arg579 / Trp631-Trp571 / Glu647-Gln563 / Glu647-^N2^Arg557 / Glu648-^N2^Arg557 / Glu648-Arg557 / Arg557-Glu654 / Lys655-Glu560 / Glu630-^N2^Arg579
**Decreasing interactions**	Lys574-Glu648 / Lys574-^N2^Leu568 / Arg579-Glu648 / Glu648-^N2^Arg579 / Glu654-Lys574
**Repulsive interactions**	Lys574-Arg633 / Lys574-^N2^Arg579 / Arg579-^N2^Arg579 / Arg579-Arg633 / Arg579-^N2^Lys574 / Lys574-^N2^Lys574 / Glu648-Glu560 / Glu648-^N2^Glu560 / Arg633-^N2^Arg579

### 3.5. Validation of computational data with experimental results

A considerable body of mutagenesis data on structure-function relationships within the HIV-1 gp41 ectodomain has been published over the years. Table C in S1 File summarizes the results of published mutagenesis studies on gp41 [20]. The HXB2 reference strain has been used as a basis for numbering individual amino acids residues. Results from the computational study are compared with experimental results. Although the computational approach did not identify some residues identified as important by mutagenesis experiments (for example Ser546) [21], it is clear that the computational data are in satisfactory qualitative agreement with the experimental information available. Indeed, with few exceptions, when the computational approach identifies a residue as contributing significantly to the conformational change free energy, such residue is also reported in mutagenesis studies as a residue that reduce cell-cell fusion and/or virus entry upon mutation. This demonstrates the reliability of the conformational transition pathway derived from the TMD simulations and the interest of our analysis. The description of the different interactions along the conformational pathway permits to understand the role of each amino acid during the physical process. Consequently, this allows us to perceive the structural and functional consequences of many amino acid substitutions in the gp41 ectodomain so they do not remain unclear from now on. Furthermore, some residues (Glu647, Glu648, Glu654 and Glu655) were detected with the computational methodology, as contributing to the TMD process, but not reported in experimental results. This suggests that the interactions created by those amino acids should be analyzed more profoundly by experiments to confirm their relevance for the conformational transition.

### 3.6. Possible sequence of events for the conformational change of HIV-1 gp41

Based on the results obtained from the TMD simulations, it is tentatively proposed below a possible sequence of events for the conformational change of HIV-1, after the exposure of the hydrophobic groove. [22–24] The stable interactions (**pillars**) identified through the computational analyses are thought to contribute to keep the two N-helices together and, consequently, to the stability of the hydrophobic groove.

**Fig 8:**
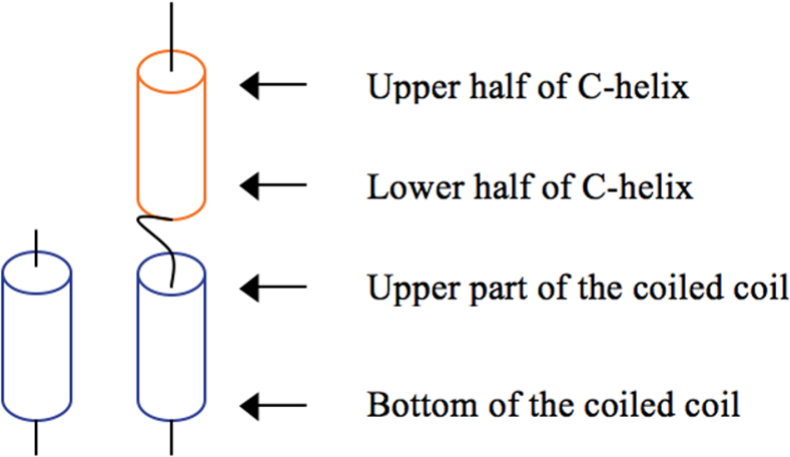
Scheme representing the different parts of the simplified model of gp41 used in this study.

Upon the formation of the intermediate, the lower half of C-helix (Fig 8) seems to be trapped by the upper part of the hydrophobic groove (**anchor points**). Then, it appears that some interactions (**guides**) start to form in order to attract the upper half of C-helix to the bottom of the hydrophobic groove. But, this is only possible if the cavity remains empty, *i*.*e*., if amino acids from N-helices do not create contacts that make them to fold over the hydrophobic groove. This undesirable episode seems to be avoided by the identified repulsive interactions (**retractors**). Furthermore, the several simulations suggest that once the guide interactions have pulled the C-helix toward the bottom of the hydrophobic groove, and that some residues from C-helix started to fit into the cavity, strong interactions (**locks**) are established in order to stabilize the accommodation of the C-helix.

### 3.7. Where should we target?

**Fig 9:**
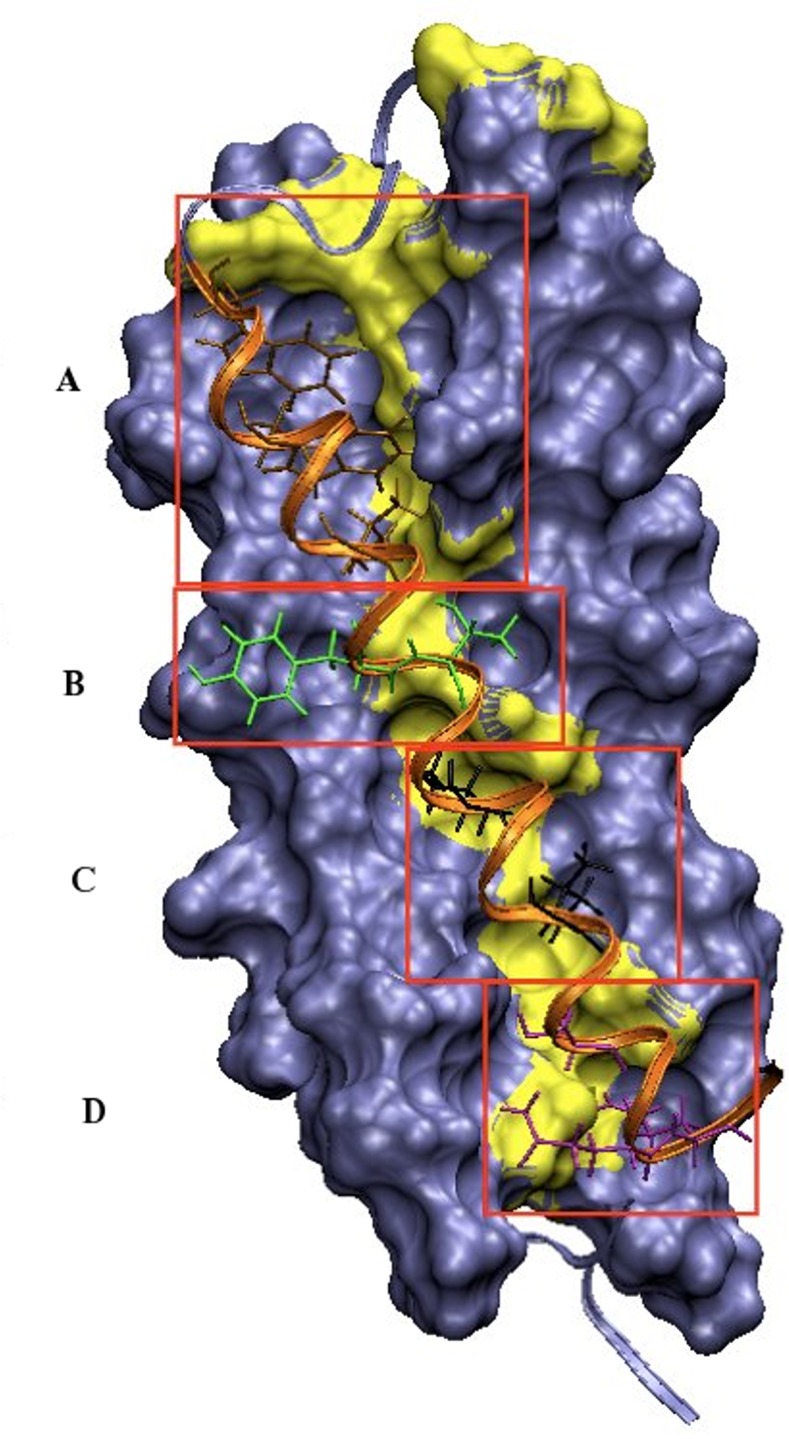
Cavities of the hydrophobic groove of HIV-1 gp41 that could be possible targets for small organic molecules. The surface represents the hydrophobic groove formed by the two N-helices and in yellow are represented the residues involved in stable interactions. C-helix is represented in orange ribbons; Residues in brown, green, black and violet correspond to the residues from C-helix that fit into the small cavity A, B, C and D, respectively.

Fig 9 summarizes four possible small cavities of the hydrophobic groove that could be targeted by small molecules in order to inhibit the conformational change of HIV-1 gp41. Each of them are filled with different ensembles of amino acids from C-helix, which makes hydrophobic contacts with residues involved in stable interactions (yellow) but also establishes other kinds of interactions (see description of main interactions section). The small cavity A corresponds to the portion of the hydrophobic groove filled with Trp628, Trp631 and Asp632. The section of the hydrophobic groove that encloses residues Tyr638 and Thr639 defines the small cavity B. The fraction that accommodates Ile642 and Ile646 corresponds to the small cavity C. And finally, the small cavity D is defined by the fragment filled with Ser649, Gln652 and Gln653.

The design of small molecules that bind to HIV-1 gp41 via one of these small cavities will probably affect, with different magnitudes, conformational transition of the protein, thus decreasing its activity. Based on the computational results, we hypothesize that targeting the small cavity A could be more efficient than targeting any of the others. Several observations sustain our hypothesis:

- The section of the hydrophobic groove corresponding to this cavity concentrates a high number of residues outlined by the computational study.
- The residues that participate in the “anchor points” (^N2^Arg579, Arg579 and Lys574) are all located at the top of the hydrophobic groove, *i*.*e*., in the neighboring environment of cavity A. And, as we mentioned in sections above, the “anchor points” seem to be crucial for the progress of the TMD pathway.
- Important locks interactions are created between residues of the cavity A and C-helix, such as Lys574-Asp632.
- Cavity A also registered the higher number of “multitask residues” when compared to the others cavities.

Analogically speaking, the conformational transition of gp41 is thought to behave like a “hinge” where the hydrophobic groove corresponds to one half and the C-helix to the other half. It is the cavity A that is closer to the axis that allows the closure of the “hinge”. Thus, if the conformational transition is interrupted close to the axis, the C-helix will be more distant from the hydrophobic groove and the possibilities to establish interactions with it will decrease.

In summary, we described an *in silico* approach that can be employed to model the large conformational transition of a HIV-1 gp41 simplified model. Several folding pathways were predicted and the approach has only been made possible by the development of specific tools and by the construction of an analysis protocol that permitted to highlight the key residues of the folding process. In addition, insights into the nature and evolution of the key interactions were provided. The results exhibited a preferred sequence of events and suggested a specific small cavity of the HIV-1 gp41 hydrophobic groove as a potential target to small molecules. The experimental mutagenesis data confirmed the TMD predictions, demonstrating the reliability of the latter. The results reported here will hopefully enable an improved design of HIV-1 entry inhibitors.

## Supporting Information

S1 File Text A in S1 File: Additional details of the preparation of the protein structures, MD simulations and targeted MD simulations; **Table A in S1 File:** Representation of the calculation of the average percentage of interaction of each possible pair of residues for the different phases, PoI(R_ij_)_Phk_, along all TMD simulations, using the free energy decomposition method; **Text B in S1 File:** Detailed main interactions established by residues that participate in the conformational change of HIV-1 gp41; **Table B in S1 File:** Correspondence between the gp160 sequence and the numbering used for TMD trajectories; **Table C in S1 File:** Table summarizing experimental gp41 mutagenesis data; **Figure A in S1 File:** Scheme illustrating the two required criteria for an intermediate to proceed to the TMD step; **Figure B in S1 File:** Plots of the evolution of all the defined leading interactions along the TMD pathway.
(PDF)
