# Supplementary material for: Striking HIV-1 Entry by Targeting HIV-1 gp41. But, Where Should We Target?
Source: PLoS One. 2016 Jan 19;11(1):e0146743. doi: 10.1371/journal.pone.0146743 (PMC4718650; doi:10.1371/journal.pone.0146743)
Supplement: S1 File — Additional details of the preparation of the protein structures, MD simulations and targeted MD simulations; Table A in S1 File: Representation of the calculation of the average percentage of interaction of each possible pair of residues for the different phases, PoI(Rij)Phk, along all TMD simulations, using the free energy decomposition method; Text B in S1 File: Detailed main interactions established by residues that participate in the conformational change of HIV-1 gp41; Table B in S1 File: Correspondence between the gp160 sequence and the numbering used for TMD trajectories; Table C in S1 File: Table summarizing experimental gp41 mutagenesis data; Figure A in S1 File: Scheme illustrating the two required criteria for an intermediate to proceed to the TMD step; Figure B in S1 File: Plots of the evolution of all the defined leading interactions along the TMD pathway. (PDF) [file pone.0146743.s001.pdf]

Supporting Information

Text A – Material and Methods (details)

Preparation of the gp41 fusogenic structure. We started from a crystallized experimental fusogenic conformation of gp41 (PDB code 1DF5) with 2.70 Å resolution. The simplified model was defined as three helices: 2 N-helices and the corresponding C-helix. Thus, the coordinates of chain B and all the residues after residue at position 38 (inclusive) of chain C were removed. The protein residues were considered to bear the charges they would have at neutral pH, i.e., Lys and Arg residues were positively charged, whereas Asp and Glu presented a negative charge. For His residues, the default neutral protonation state (HIE, with hydrogen on the delta nitrogen) in AMBER9 was used. The X-ray structure was then subjected to a minimization carried out using the SANDER module of AMBER 9.0 with the FF03 Cornell et al. force field [1]. The generalized Born (GB) implicit solvent model was used and the minimization process started with 500 steps of steepest descent followed by 2000 steps of conjugate gradient. Second, in order to reach stabilization of the gp41 fusogenic structure, a conventional molecular dynamics (MD) simulation was carried out at 300 K, using Langevin dynamics [2] with a collision frequency of 1.0 ps^{-1}. Shake constraints were applied to all bonds involving hydrogen atoms [3]. The time step for integrating the equations of motion was set to 2 fs. The addition of two distance restraints was required in order to maintain the two N-helices together once we were using a simplified model. A distance of 11.10 Å and 10.70 Å was set between the first and the last Cα atoms (Val549-^{N2}Val549 and Arg579-^{N2}Arg579) of the two N-helices respectively, with a force constant of 10 $kcal\cdot mol^{-1}$. All intermolecular interactions were treated with a cutoff distance of 20 Å. The length of the production run was 3000 ps and the RMSD values of Cα atoms converged for the last nanosecond of the trajectory. The average structure of the last nanosecond was taken as the gp41 fusogenic representative conformation.

Generation of the gp41 nonfusogenic structure. We assumed that in the nonfusogenic conformation of gp41 the linker between helices N and C would appear as a beta-sheet, which is known to be a classic conformation for a peptide backbone. This change was made with the help of Biopolymer module of Sybyl [4] by assigning a beta-sheet conformation to the six residues SGGRGG link. The protonation of protein residues and the minimizations were then performed as described for the generation of the gp41 fusogenic structure. Firstly, we minimized the peptide residues keeping the

other protein atoms constrained by weak harmonic restraints. Then, we performed 500 steps of steepest descent followed by 2000 steps of conjugate gradient minimizations of the entire system.

Classical molecular dynamics simulations. The molecular dynamics simulations were carried out at 300 K using Langevin dynamics [2] with a collision frequency of 1.0 ps^{-1}. Shake constraints were applied to all bonds involving hydrogen atoms [3]. The time step for integrating the equations of motion was set to 2 fs. The addition of two distance restraints was required in order to maintain the two N-helices together once we were using a simplified model. A distance of 11.10 Å and 10.70 Å was set between the first and the last Cα atoms (Val549-^{N2}Val549 and Arg579-^{N2}Arg579) of the two N-helices respectively, with a force constant of 10 $kcal \cdot mol^{-1}$. All intermolecular interactions were treated with a cutoff distance of 20 Å. The length of the production run was 1000 ps.

Definition of the criteria of selection. The criteria of selection were based on the direction that the C-helix took and the angle that it formed with its linked N-helix, after the MD run (Figure A). We assumed that the C-helix moved into the right direction if placed into an area limited by an angle of 90º relative to the axis along the N-helices (Figure Aa). The other criterion of selection corresponds to the degree of folding presented by the intermediate and we assumed that a suitable degree of folding was reached when the C-helix formed an angle with its linked N-helix lower than 120º (Figure Ab).

Data analysis – free energy decomposition. A set of snapshots was extracted (every 5 ps) along the entire TMD trajectory. For each snapshot, gas-phase energies were then calculated by summing contributions from internal energies (bond, angle and torsional angle), electrostatic energies, and van der Waals energies using the FF03 Cornell *et al.* force field with no cut-off; 1-4 electrostatic and 1-4 van der Waals energies were added to electrostatic and van der Waals contributions, respectively. The polar contribution to free energy of solvation was calculated by applying the analytical generalized Born approximation and a dielectric constant, $\varepsilon = 80$, was used for the solvent.

Data analysis – evolution of the main interactions over time.

The free energy decomposition on a pairwise per-residue basis was applied, at different phases of the trajectory, to the identified key interactions. Hence, each TMD simulation was divided into periods of 100 ps and the free energy decomposition calculation was performed for each of those time periods. The procedure was repeated for all the analysed TMD pathways and, finally, a percentage of interaction was obtained for each pair of residues every time period of 100 ps and for each TMD

simulation, $PoI^{R_{ij}}_{Ph_k,T_i}$. Then, an average percentage of interaction was calculated for each pair of residues for the different phases of the TMD trajectories, $PoI(R_{ij})_{Phk}$.

Noteworthy, the TMD trajectories do not present the same number of phases. Indeed, the time of each simulation is dependent of the intermediate structure from which the TMD simulation started. Consequently, some TMD pathways were performed on about 700 ps, while others took about 1200 ps to converge to the target structure. Therefore, the different TMD simulations were aligned by the ending time and an average percentage of interaction was calculated only from the first time period common to all paths and until the last, schematized as followed:

Table A: Explanatory representation of the calculation of the average percentage of interaction of each possible pair of residues for the different phases, $PoI(R_{ij})_{Phk}$, along all TMD simulations, using the free energy decomposition method.

Interaction issued from the 2nd step of analysis (R_i ,R_j)					
Phase (Ph_k)	TMD simulation (T) 1	2	3	...	$PoI(R_{ij})_{Phk}$ of the "Average pathway"
**1:** 1 – 100 ps	$PoI^{R_{ij}}_{Ph_1,T_1} = \frac{\Delta G^{R_{ij}}_{Ph_1,T_1}}{\Delta G^{R_{ij}}_{T_1}}$	—	—	...	—
**2:** 101 – 200 ps	$PoI^{R_{ij}}_{Ph_2,T_1}$	—	—	...	—
**3:** 201 – 300 ps	$PoI^{R_{ij}}_{Ph_3,T_1}$	—	—	...	—
**4:** 301 – 400 ps	$PoI^{R_{ij}}_{Ph_4,T_1}$	—	—	...	—
**5:** 401 – 500 ps	$PoI^{R_{ij}}_{Ph_5,T_1}$	$PoI^{R_{ij}}_{Ph_5,T_2}$	—	...	—
**6:** 501 – 600 ps	$PoI^{R_{ij}}_{Ph_6,T_1}$	$PoI^{R_{ij}}_{Ph_6,T_2}$	—	...	—
**7:** 601 – 700 ps	$PoI^{R_{ij}}_{Ph_7,T_1}$	$PoI^{R_{ij}}_{Ph_7,T_2}$	$PoI^{R_{ij}}_{Ph_7,T_3}$	...	$PoI(R_{ij})_{Ph7} = \frac{\sum_{T=1}^{n} PoI^{R_{ij}}_{Ph_7}}{\sum_{1}^{n} T}$
...	...	...	...	...	...
**12:** 1101 – 1200 ps	$PoI^{R_{ij}}_{Ph_{12},T_1}$	$PoI^{R_{ij}}_{Ph_{12},T_2}$	$PoI^{R_{ij}}_{Ph_{12},T_3}$	...	$PoI(R_{ij})_{Ph12} = \frac{\sum_{T=1}^{n} PoI^{R_{ij}}_{Ph_{12}}}{\sum_{1}^{n} T}$

The percentage of interaction by phase, $PoI_{Ph_k,T_i}^{R_{ij}}$, of a pair of residues (R_i, R_j) during a phase, Ph_k, of a TMD simulation, T_i, is obtained by the quotient between the pairwise contribution of the residue R_i to the free energy, resulting from its interaction with the residue R_j during the time period corresponding to the respective phase, $\Delta G_{Ph_k,T_i}^{R_{ij}}$, and the total pairwise contribution of the contact (R_i, R_j) to the free energy along the total time simulation, $\Delta G_{Ti}^{R_{ij}}$. Then, the average percentage of interaction by phase was calculated for each pair of residues, $PoI(R_{ij})_{Phk}$, using the $PoI_{Ph_k,T_i}^{R_{ij}}$ values along the different TMD trajectories.

As mentioned above, the different TMD pathways did not present the same time of simulation. Thus, the $PoI(R_{ij})_{Phk}$ was determined only for the time periods common to all the trajectories after aligned by the last phase. A total of 12 different phases was obtained since the longest TMD simulation was performed for about 1200 ps and the shorter one for about 700 ps. Therefore, a value of $PoI_{Ph_k,T_i}^{R_{ij}}$ was calculated for the last 6 phases of all the TMD trajectories corresponding, approximately, to the time period of 600-1200 ps. The results were presented as a plot of $PoI(R_{ij})_{Phk}$ for each pairwise interaction (R_i, R_j) over the different phases of the "average pathway".

Text B – Results and Discussion: description of main interactions

Gln552: Both methods indicated that this residue establishes a main interaction with ^{N2}**Gln551**. The interaction is mainly due to hydrogen bonds and electrostatic interactions between the polar moieties of the two glutamate side-chains. We also verified smaller interactions with ^{N2}**Gln552**, ^{N2}**Asn554** and ^{N2}**Leu555**.

Leu556: This residue was only outlined with the free energy decomposition method. We observed a major interaction with ^{N2}**Leu555** mainly originated by van der Waals contacts and a favorable polar solvation free energy. Leu11 creates additional smaller interactions with spatially adjacent residues such as ^{N2}**Gln551** and ^{N2}**Asn554**.

Arg557: This residue was identified only by the H_b/E_i method. A strong interaction was observed with **Glu654**, mainly originated by a salt bridge between the positively charged group of Arg557 and the negatively charged side-chain of Glu654. This interaction corresponds to one N-C helices interaction, which suggests that this interhelical contact is critical for keeping the stability of the trimer-of-hairpins.

Ile559: This residue was pointed out by the two methods and presents an obvious main interaction (hydrogen bond) with ^{N2}**Gln562**, which corresponds to a N-N contact. Smaller interactions (mainly van der Waals contacts) with spatially adjacent residues (^{N2}**Ile559** and ^{N2}**Leu555**) were also observed.

Glu560: This amino acid was outlined by the H_b/E_i method and it establishes a predominant interaction (salt bridge between the charged side-chains) with **Lys655**. As for Arg557-Glu654 interaction, this contact also corresponds to an interaction between the N- and C-helices and seems to contribute to the stability of the trimer-of-hairpins.

Gln562, Gln563: These two amino acids can be put together since they present the same behavior and were both pointed out by the two methods. A strong interaction with ^{N2}**Gln562** was observed and is mainly due to electrostatic contacts between the polar groups of both residues involved.

Leu566: This residue was identified by the free energy decomposition method only. A major interaction, mainly due to hydrophobic contacts, with ^{N2}**Leu565** and ^{N2}**Leu566** was detected.

Val570: This residue was outlined by the free energy decomposition analysis and a major interaction with **^{N2}Thr569** was identified. The favorable contact is mostly due to van der Waals interactions established between the side-chains of the amino acids. Two more significant interactions were detected with its neighbor residues **^{N2}Leu565** and **^{N2}Leu568**. These two interactions are also mostly due to van der Waals contacts.

Ile573: This amino acid was pointed out by the free energy decomposition analysis. We detected several main interactions with spatially adjacent amino acids such as **^{N2}Thr569**, **^{N2}Ile573** and **^{N2}Leu576**. All these favorable interactions are principally due to van der Waals and hydrophobic contacts.

Trp631: This residue was highlighted by the two methods. Like for Trp628, this amino acid mostly contributes to the stability of the trimer-of-hairpins since it creates N-C interactions with several N-helices residues: **^{N2}Leu568,** **^{N2}Arg579**, **Trp571**, **Lys574**, **Gln577**, **^{N2}Trp571** and **^{N2}Gln575**. The contacts made with the last four residues are predominantly interactions that contribute to the stability of the trimer upon the large movement of gp41 is completed. In fact, these four amino acids, in addition with ^{N2}Leu568, form a hydrophobic cavity where Trp631 accommodates. ^{N2}Arg579 and Trp571 are thought to contribute as guides during the TMD trajectories since they are not in the close neighbouring environment.

Asp632: The two methods outlined this residue. Asp632 seems to be crucial for the stability of the trimer-of-hairpin with a favorable contribution originated by the salt bridge established with **Lys574**. This contact is undoubtedly pointed out as an important one by the two methods. However, both techniques also revealed other significant interactions (long range interactions) with **^{N2}Arg579** and **Arg579**, which are thought to act as "anchor" points. Repulsive interactions are also detected, with Glu560 and ^{N2}Glu560 and are discussed in Section 3.4 of the manuscript.

Glu647: This amino acid was only highlighted by the H_b/E_i method and was found to form a significant interaction with **^{N2}Arg557** and weaker ones with **^{N2}Gln551** and **^{N2}Gln562**. These contacts contribute favorably to the conformational change free energy by the mean of electrostatic interactions between their polar group and the carboxylate moiety of Glu647.

Table B: Residue sequence, of the three helices of the simplified model of gp41. The numbering of the residues is provided according to the pdb used in this study (left column) and to the gp160 sequence (right column). The amino acids issued from the first phase of the analysis are in bold.

	N2			C			N1	
**Residue number (pdb)**	**Residue**	**Residue number (gp160)**	**Residue number (pdb)**	**Residue**	**Residue number (gp160)**	**Residue number (pdb)**	**Residue**	**Residue number (gp160)**
**102**	**Arg**	**579**				**34**	**Arg**	**579**
101	Ala	578				33	Ala	578
100	Gln	577				**32**	**Gln**	**577**
**99**	**Leu**	**576**	**41**	**Trp**	**628**	31	Leu	576
98	Gln	575	42	Met	629	30	Gln	575
97	Lys	574	43	Glu	630	**29**	**Lys**	**574**
96	Ile	573	**44**	**Trp**	**631**	**28**	**Ile**	**573**
95	Gly	572	**45**	**Asp**	**632**	27	Gly	572
94	Trp	571	46	Arg	633	26	Trp	571
93	Val	570	47	Glu	634	**25**	**Val**	**570**
**92**	**Thr**	**569**	48	Ile	635	24	Thr	569
91	Leu	568	49	Asn	636	23	Leu	568
90	Gln	567	50	Asn	637	22	Gln	567
89	Leu	566	51	Tyr	638	**21**	**Leu**	**566**
**88**	**Leu**	**565**	52	Thr	639	20	Leu	565
87	His	564	53	Ser	640	19	His	564
86	Gln	563	54	Leu	641	**18**	**Gln**	**563**
**85**	**Gln**	**562**	55	Ile	642	**17**	**Gln**	**562**
84	Ala	561	56	His	643	16	Ala	561
83	Glu	560	57	Ser	644	**15**	**Glu**	**560**
**82**	**Ile**	**559**	58	Leu	645	**14**	**Ile**	**559**
81	Ala	558	59	Ile	646	13	Ala	558
**80**	**Arg**	**557**	**60**	**Glu**	**647**	**12**	**Arg**	**557**
79	Leu	556	**61**	**Glu**	**648**	**11**	**Leu**	**556**
**78**	**Leu**	**555**	62	Ser	649	10	Leu	555
77	Asn	554	63	Gln	650	9	Asn	554
76	Asn	553	64	Asn	651	8	Asn	553
75	Gln	552	65	Gln	652	**7**	**Gln**	**552**
**74**	**Gln**	**551**	66	Gln	653	6	Gln	551
73	Gln	550	**67**	**Glu**	**654**	5	Gln	550
72	Val	549	**68**	**Lys**	**655**	4	Val	549
71	Ile	548				3	Ile	548
70	Gly	547				2	Gly	547
69	Ser	546				1	Ser	546

Table C: Summary of experimental gp41 mutagenesis data. Adapted from [5]

Residue number (gp160)[1]	Subtitution	Isolate	Cell-cell fusion[2]	Virus entry[3]	Reference
^{N2}Gln551	A	HXB2	++	+	[6, 7]
Gln552	L	HXB2	-		[8]
^{N2}Leu555	G	HXB2	-		[8]
	A	BH8	-		[9]
Leu556	A	HXB2	-	+	[6, 10]
Arg557; ^{N2}Arg557	A	HXB2		+	[7]
Ile559; ^{N2}Ile559	A	BH8	-		[9]
Glu560	G	ELI1	+		[11]
Gln562; ^{N2}Gln562	A	BH8	-		[9]
Gln563	A	HXB2		++	[12]
	E			-	
	M			-	
	R			+	
^{N2}Leu565	A	HXB2	-	-	[6, 10]
Leu566	G	HXB2	-	+	[8]
	P	HXB2	-	-	[13, 14]
	A	BH8	-		[9]
^{N2}Thr569	A	BH8	-		[9]
Val570	R	HXB2	-	-	[15]
	D			-	
	E	HXB2		-	[12]
	G			-	
	A	HXB2	-	-	[6, 10]
Ile573	A	HXB2	+		[16]
	G		-		
	E		-		
	D		-		
	S		-		
Lys574	R	BH8	+		[17]
^{N2}Leu576	P	HXB2	-		[13]
	A	BH8	-		[9]
Gln577	R	HXB2		-	[15]
	E		+	+	
Arg579; ^{N2}Arg579	G	HXB2		-	[12]
	A	HXB2	-		[10]
Trp628	A	HXB2		-	[15]
Trp631	A	HXB2	-		[18]
Asp632	N	BH10	-		[19]

[1] The residue numbering based on HXB2 gp160 could correspond to a residue of any of the three N-helices. However in our study the same residue appertaining to different N-helices are distinguished. That is why, we have situations where one residue (with numbering based on gp160) correspond to two residues in our study, one from each N-helices.

[2] As assessed by syncitium formation or reporter gene assays. -, fusion lower than 3% of WT; +, fusion between 3 and 30% of WT; ++ fusion greater than 30% of WT.

[3] As assessed by various assays (replication complementation, use of reporter genes, p24 production). -, entry lower than 3% of WT; +, entry between 3 and 30% of WT; ++, entry greater than 30% of WT.

References SI:

1. Yang L, Han CH, Hsieh MJ, Wang J, Duan Y, Cieplak P, et al. Biophysiscs. 2006;110:13166.
2. Uberuaga BP, Anghel M, Voter AF. Synchronization of trajectories in canonical molecular-dynamics simulations: observation, explanation, and exploitation. J Chem Phys. 2004;120(14):6363-74. Epub 2004/07/23. doi: 10.1063/1.1667473. PubMed PMID: 15267525.
3. Ryckaert J-P, Ciccotti G, Berendsen HJC. Numerical integration of the cartesian equations of motion of a system with constraints: Molecular Dynamics of n-alkanes. J Comput Phys. 1977;23:327-41.
4. Sybyl 7.3, Tripos Software, St. Louis, USA. Sybyl 7.3, Tripos Software, St. Louis, USA.
5. Sanders RW, Korber B, Lu M, Berkhout B, Moore JP. HIV Sequence Compendium, 2002. Theoretical Biology and Biophysics Group, Los Alamos National Laboratory, Los Alamos, NM. 2002.
6. Follis KE, Larson SJ, Lu M, Nunberg JH. Genetic evidence that interhelical packing interactions in the gp41 core are critical for transition of the human immunodeficiency virus type 1 envelope glycoprotein to the fusion-active state. Journal of virology. 2002;76(14):7356-62. PubMed PMID: 12072535; PubMed Central PMCID: PMC136323.
7. Sen J, Yan T, Wang J, Rong L, Tao L, Caffrey M. Alanine scanning mutagenesis of HIV-1 gp41 heptad repeat 1: insight into the gp120-gp41 interaction. Biochemistry. 2010;49(24):5057-65. doi: 10.1021/bi1005267. PubMed PMID: 20481578.
8. Cao J, Bergeron L, Helseth E, Thali M, Repke H, Sodroski J. Effects of amino acid changes in the extracellular domain of the human immunodeficiency virus type 1 gp41 envelope glycoprotein. Journal of virology. 1993;67(5):2747-55. PubMed PMID: 8474172; PubMed Central PMCID: PMC237598.
9. Poumbourios P, Wilson KA, Center RJ, El Ahmar W, Kemp BE. Human immunodeficiency virus type 1 envelope glycoprotein oligomerization requires the gp41 amphipathic alpha-helical/leucine zipper-like sequence. Journal of virology. 1997;71(3):2041-9. PubMed PMID: 9032336; PubMed Central PMCID: PMC191291.
10. Lu M, Stoller MO, Wang S, Liu J, Fagan MB, Nunberg JH. Structural and functional analysis of interhelical interactions in the human immunodeficiency virus type 1 gp41 envelope glycoprotein by alanine-scanning mutagenesis. Journal of virology. 2001;75(22):11146-56. doi: 10.1128/JVI.75.22.11146-11156.2001. PubMed PMID: 11602754; PubMed Central PMCID: PMC114694.
11. Kozak SL, Platt EJ, Madani N, Ferro FE, Jr., Peden K, Kabat D. CD4, CXCR-4, and CCR-5 dependencies for infections by primary patient and laboratory-adapted isolates of human immunodeficiency virus type 1. Journal of virology. 1997;71(2):873-82. PubMed PMID: 8995603; PubMed Central PMCID: PMC191134.
12. Weng Y, Yang Z, Weiss CD. Structure-function studies of the self-assembly domain of the human immunodeficiency virus type 1 transmembrane protein gp41. J Virol. 2000;74(11):5368-72. Epub 2000/05/09. PubMed PMID: 10799616; PubMed Central PMCID: PMC110894.
13. Chen SS. Functional role of the zipper motif region of human immunodeficiency virus type 1 transmembrane protein gp41. Journal of virology. 1994;68(3):2002-10. PubMed PMID: 7509005; PubMed Central PMCID: PMC236667.
14. Chen SS, Lee CN, Lee WR, McIntosh K, Lee TH. Mutational analysis of the leucine zipper-like motif of the human immunodeficiency virus type 1 envelope transmembrane glycoprotein. Journal of virology. 1993;67(6):3615-9. PubMed PMID: 8497069; PubMed Central PMCID: PMC237711.

15. Weng Y, Weiss CD. Mutational analysis of residues in the coiled-coil domain of human immunodeficiency virus type 1 transmembrane protein gp41. Journal of virology. 1998;72(12):9676-82. PubMed PMID: 9811701; PubMed Central PMCID: PMC110477.
16. Dubay JW, Roberts SJ, Brody B, Hunter E. Mutations in the leucine zipper of the human immunodeficiency virus type 1 transmembrane glycoprotein affect fusion and infectivity. Journal of virology. 1992;66(8):4748-56. PubMed PMID: 1629954; PubMed Central PMCID: PMC241301.
17. McInerney TL, El Ahmar W, Kemp BE, Poumbourios P. Mutation-directed chemical cross-linking of human immunodeficiency virus type 1 gp41 oligomers. Journal of virology. 1998;72(2):1523-33. PubMed PMID: 9445056; PubMed Central PMCID: PMC124634.
18. Vidal N, Peeters M, Mulanga-Kabeya C, Nzilambi N, Robertson D, Ilunga W, et al. Unprecedented degree of human immunodeficiency virus type 1 (HIV-1) group M genetic diversity in the Democratic Republic of Congo suggests that the HIV-1 pandemic originated in Central Africa. Journal of virology. 2000;74(22):10498-507. PubMed PMID: 11044094; PubMed Central PMCID: PMC110924.
19. Perrin C, Fenouillet E, Jones IM. Role of gp41 glycosylation sites in the biological activity of human immunodeficiency virus type 1 envelope glycoprotein. Virology. 1998;242(2):338-45. doi: 10.1006/viro.1997.9016. PubMed PMID: 9514971.

Figure A: Scheme illustrating the two required criteria (shadow areas) so the intermediates, issued from the MD runs, are eligible to proceed to the TMD run. a) Top view of gp41 fusogenic structure. The shadow area represents the orthogonal angle, between C-helix and the axis along the two N-helices. b) Side view of the gp41 fusogenic structure. The shadow area corresponds to the angle formed between the C-helix and its linked N-helix.

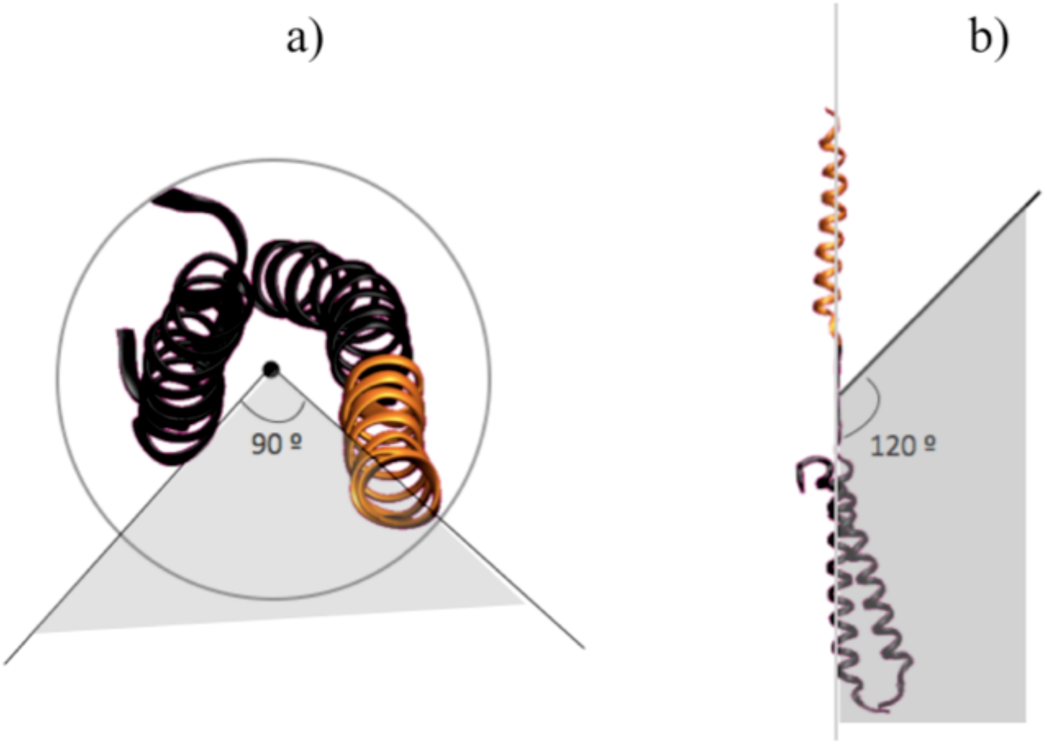

Figure B - Plots representing the evolution of the main interactions between residues along the TMD pathway. $PoI_{Ph_k}^{R_i,R_j}$ corresponds to the percentage of interaction (PoI) between residues, R_i and R_j, along the different phases (Ph_k) of the conformational change.

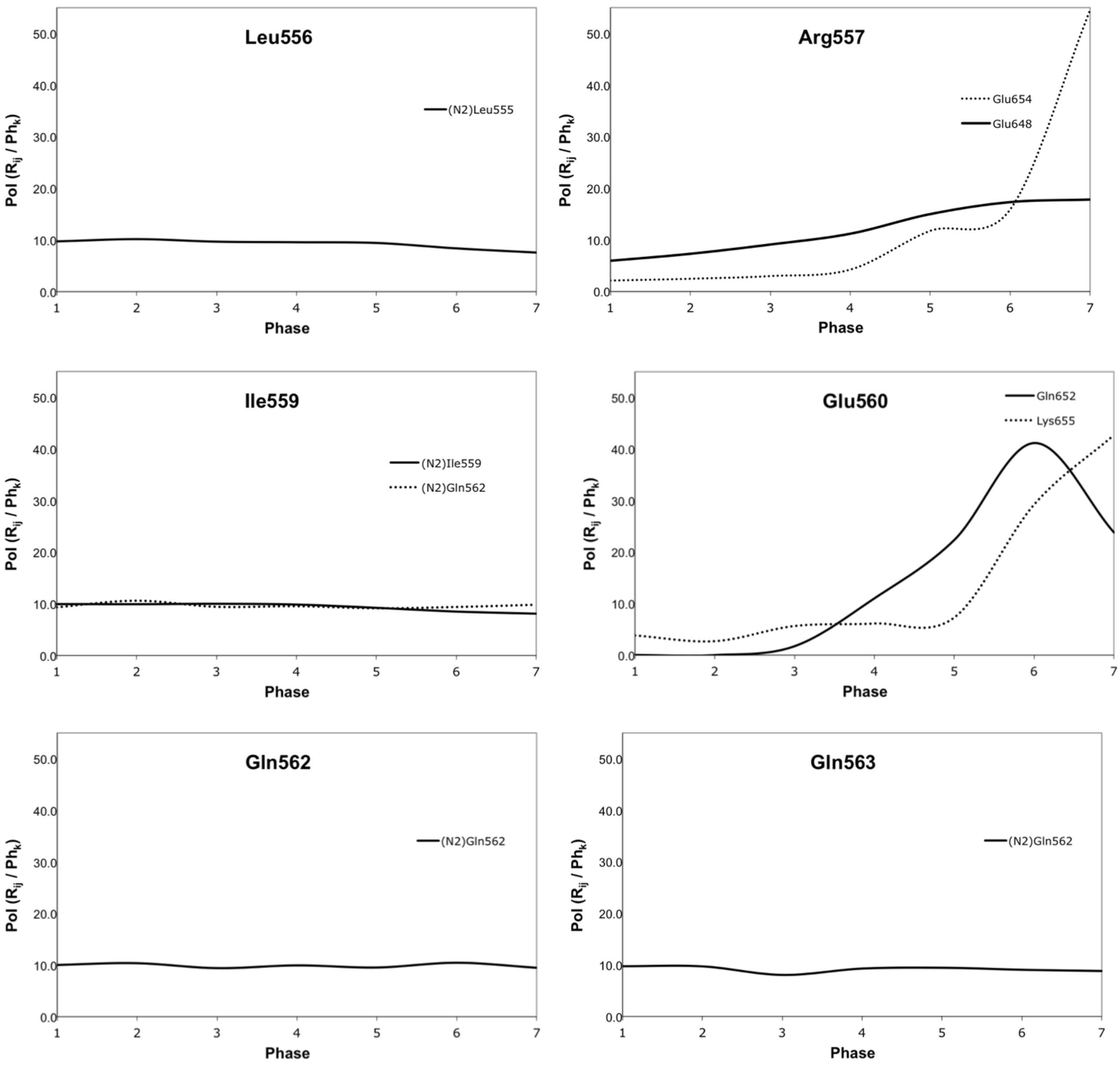

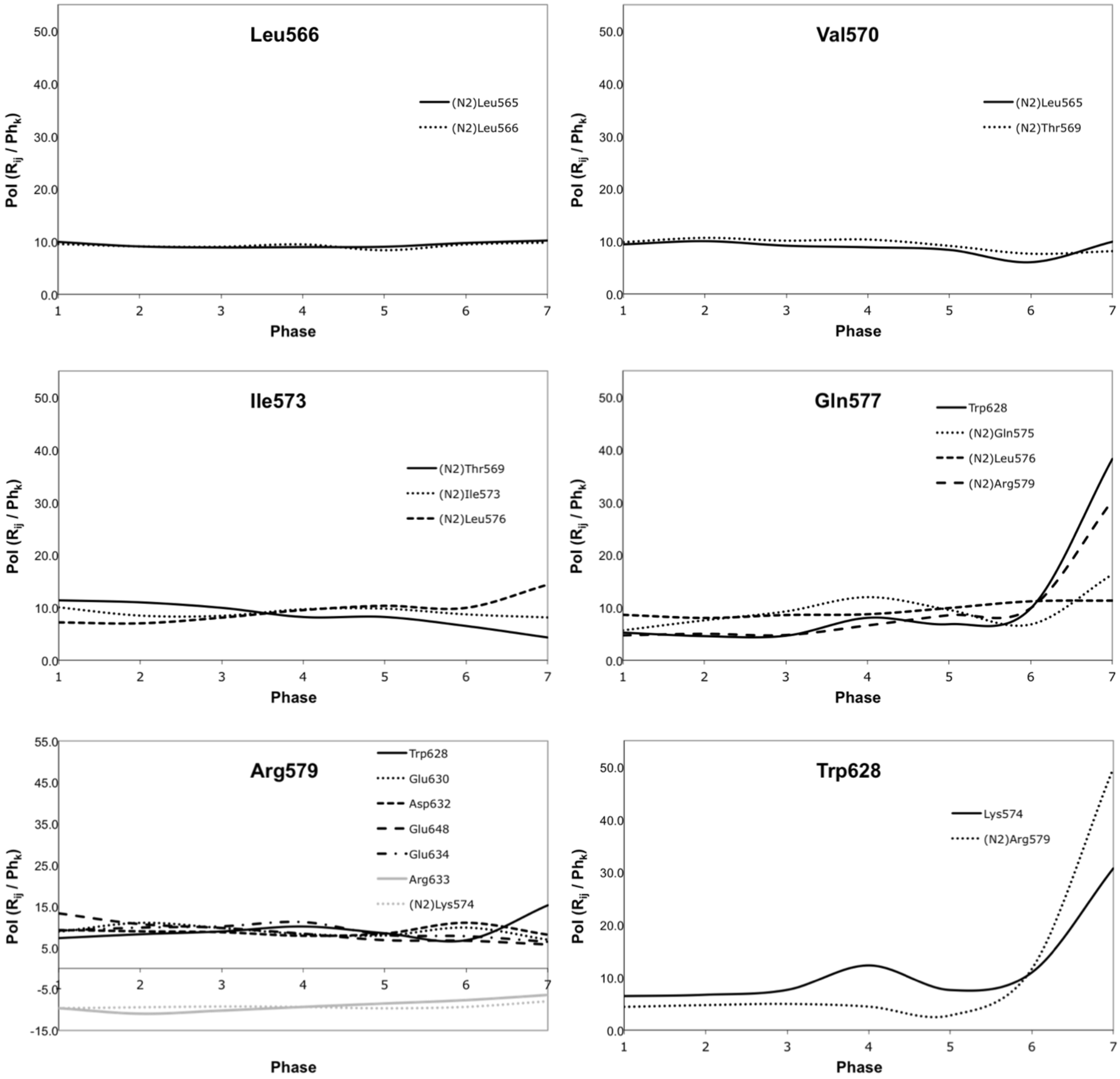
Leu566
(N2)Leu565
(N2)Leu566
Val570
(N2)Leu565
(N2)Thr569
Ile573
(N2)Thr569
(N2)Ile573
(N2)Leu576
Gln577
Trp628
(N2)Gln575
(N2)Leu576
(N2)Arg579
Arg579
Trp628
Glu630
Asp632
Glu648
Glu634
Arg633
(N2)Lys574
Trp628
Lys574
(N2)Arg579
Pol (R_{ij} / Ph_k)
Phase

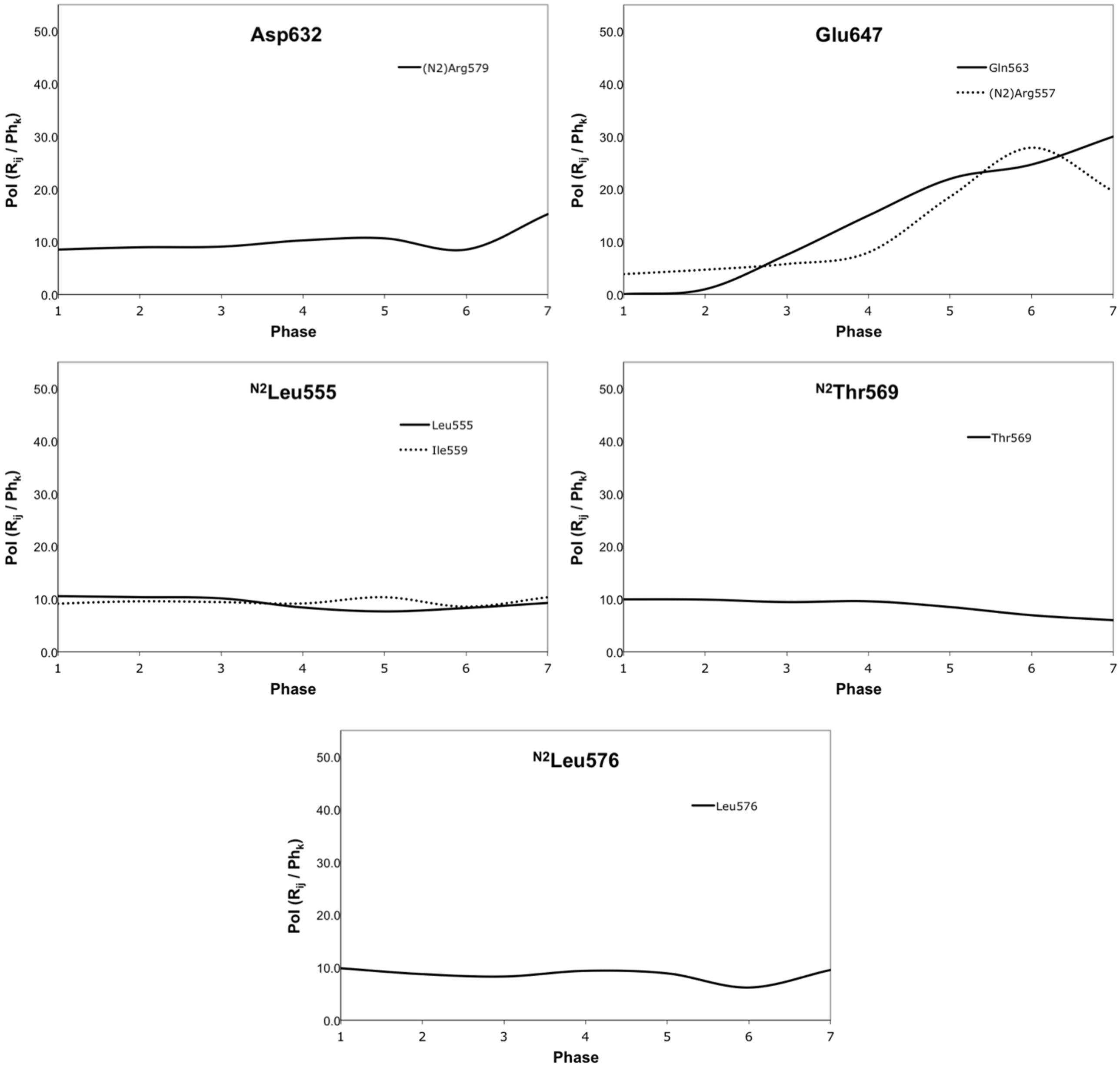
Asp632
(N2)Arg579
Glu647
Gln563
(N2)Arg557
N2Leu555
Leu555
Ile559
N2Thr569
Thr569
N2Leu576
Leu576
Pol (Rij / Phk)
Phase
0.0
10.0
20.0
30.0
40.0
50.0
1
2
3
4
5
6
7
